# Self-fertility in *Chromocrea spinulosa* is a consequence of direct repeat-mediated loss of *MAT1-2*, subsequent imbalance of nuclei differing in mating type, and recognition between unlike nuclei in a common cytoplasm

**DOI:** 10.1371/journal.pgen.1006981

**Published:** 2017-09-11

**Authors:** Sung-Hwan Yun, Hee-Kyoung Kim, Theresa Lee, B. Gillian Turgeon

**Affiliations:** 1 Department of Medical Biotechnology, Soonchunhyang University, Asan, Chungnam, Republic of Korea; 2 Microbial Safety Team, National Institute of Agricultural Science, Rural Development Administration, Wanju, Jeonbuk, Republic of Korea; 3 Plant Pathology & Plant-Microbe Biology Section, School of Integrative Plant Science, Cornell University, Ithaca, NY, United States of America; Duke University Medical Center, UNITED STATES

## Abstract

The filamentous fungus *Chromocrea spinulosa* (*Trichoderma spinulosum*) exhibits both self-fertile (homothallic) and self-sterile (heterothallic) sexual reproductive behavior. Self-fertile strains produce progeny cohorts that are 50% homothallic, 50% heterothallic. Heterothallic progeny can mate only with homothallic strains, and progeny also segregate 50% homothallic, 50% heterothallic. Sequencing of the mating type (*MAT*) region of homothallic and heterothallic strains revealed that both carry an intact *MAT1-1* locus with three *MAT1-1* genes (*MAT1-1-1*, *MAT1-1-2*, *MAT1-1-3*), as previously described for the Sordariomycete group of filamentous fungi. Homothallic strains, however, have a second version of *MAT* with the *MAT1-2* locus genetically linked to *MAT1-1*. In this version, the *MAT1-1-1* open reading frame is split into a large and small fragment and the truncated ends are bordered by 115bp direct repeats (DR). The *MAT1-2-1* gene and additional sequences are inserted between the repeats. To understand the mechanism whereby *C*. *spinulosa* can exhibit both homothallic and heterothallic behavior, we utilized molecular manipulation to delete one of the DRs from a homothallic strain and insert *MAT1-2* into a heterothallic strain. Mating assays indicated that: i) the DRs are key to homothallic behavior, ii) looping out of *MAT1-2-1 via* intra-molecular homologous recombination between the DRs in self-fertile strains results in two nuclear types in an individual (one carrying both *MAT1-1* and *MAT1-2* and one carrying *MAT1-1* only), iii) self-fertility is achieved by inter-nuclear recognition between these two nuclear types before meiosis, iv) the two types of nuclei are in unequal proportion, v) having both an intact *MAT1-1-1* and *MAT1-2-1* gene in a single nucleus is not sufficient for self-fertility, and vi) the large truncated *MAT1-1-1* fragment is expressed. Comparisons with *MAT* regions of *Trichoderma reesei* and *Trichoderma virens* suggest that several crossovers between misaligned parental *MAT* chromosomes may have led to the *MAT* architecture of homothallic *C*. *spinulosa*.

## Author summary

Fungi employ one of two mating tactics for sexual reproduction: self-sterile/heterothallic species can mate only with a genetically distinct partner while self-fertile/homothallic species do not require a partner. In ascomycetes, sexual reproduction is controlled by master regulators encoded by the mating-type (*MAT*) locus. The architecture of *MAT* differs in heterothallic *versus* homothallic species; heterothallics carry one of two forms (*MAT1-1* or *MAT1-2*) per nucleus, whereas most homothallics carry both *MAT* forms in a single nucleus. There are intriguing exceptions. For example, the yeast models, *Saccharomyces cerevisiae*, and *Schizosaccharomyces pombe* undergo reversible *MAT* switching, not demonstrated in filamentous fungi. Here, we describe the mating mechanism in *Chromocrea spinulosa* (*Trichoderma spinulosum*), a filamentous ascomycete that exhibits both homothallic and heterothallic behavior. Self-fertile strains produce progeny cohorts that are 50% homothallic, 50% heterothallic. Self-sterile strains can mate only with homothallic strains, and when this occurs, homothallic and heterothallic progeny are also produced in a 1:1 ratio. By *MAT* sequencing and manipulation, we discovered unique *MAT* architecture and determined that self-fertility is achieved by deletion of *MAT1-2* from most homothallic nuclei and subsequent inter-nuclear recognition between the resulting two, unevenly present, nuclear types in a common cytoplasm.

## Introduction

Most fungi use one of two sexual reproductive strategies, *i*.*e*., heterothallism (self-sterility) or homothallism (self-fertility). A heterothallic fungus requires a genetically distinct partner to complete the sexual process, whereas a homothallic one does not require a partner [1]. In ascomycetes, sexual reproduction of both heterothallic and homothallic species is controlled by a single master regulatory locus called the mating-type (*MAT*) locus [1]. All heterothallic ascomycetes examined to date carry one of two *MAT* forms (idiomorphs [2]) per nucleus, that encode apparently unrelated, but, in fact, ancestrally related transcription factors [3]. Most homothallic species carry both *MAT* forms in a single nucleus [1,4,5]. There is a significant difference in the use of the term “homothallism” in filamentous fungi *versus* in yeasts such as *Saccharomyces cerevisiae*. Homothallism in the latter refers to a change in mating type/identity of some cells within a culture of a formerly uniform mating identity, followed by mating of “switched” with “unswitched” cells [6]. In *S*. *cerevisiae*, three *MAT* loci (one active and two silent loci containing opposite *MAT* genes) are linked on the same chromosome. Switching is achieved by homologous intramolecular recombination-mediated replacement of the active copy at *MAT* with a formerly silent copy of opposite mating type [6,7]. Methylotrophic yeasts, such as *Hansenula polymorpha* and *Pichia pastoris* have a simpler switching system, achieved by an inversion between two *MAT* loci (one active, one silent), mediated by inverted repeats located nearby [8,9]. This mechanism silences the formerly active *MAT*, thus altering mating type [10]. Although switching of mating type observed in the yeasts has not been demonstrated in filamentous ascomycetes, mating type instability has been reported for several, including *Chromocrea* (= *Hypocrea*) *spinulosa* (*Trichoderma spinulosum*) [11] (S1 Fig), *Glomerella cingulata* [12], various *Ceratocystis* species [13,14], *Sclerotinia trifoliorum* [15], *Fusarium subglutinans* [16], and *Botrytinia fuckeliana* [17]. Interestingly, only unidirectional switching of mating type has been observed in these cases [18]. For example, selfing of *C*. *spinulosa* and *S*. *trifoliorum*, both of which are assumed to be haploid, yields equal ratios of large and small ascospores (8:8 in *C*. *spinulosa* and 4:4 in *S*. *trifoliorum*) in a single ascus; mating ability segregates with size. Colonies derived from large spores (L) are self-fertile whereas those from small spores (S) are self-sterile; selfing of the former results in asci containing, again, large and small spores in equal ratio [11,19]. Unlike the well-described mechanism of mating-type switching characterized for e.g., *S*. *cerevisiae*, the molecular details of unidirectional switching in fungi such as *C*. *spinulosa*, *Ceratocystis fimbriata*, and *S*. *trifoliorum* are unclear, however deletion of *MAT1-2-1* is involved in all three cases [20–23].

To explore this question in depth in *C*. *spinulosa*, we employed sequence and expression analyses of the *MAT* region, plus functional manipulation of genes. We present evidence supporting our hypothesis that, in order to self, homothallic strains must generate two versions of the *MAT* locus. One is the well-described *MAT1-1* locus of Sordariomycetes encoding three genes, *MAT1-1-1*, *MAT1-1-2* and *MAT1-1-3*, while the other is an altered *MAT1-1* locus in which *MAT1-2* is situated between truncated fragments of *MAT1-1-1* (*MAT1-1*;*MAT1-2*). An 115 bp stretch of the *MAT1-1-1* coding sequence is duplicated in each truncated *MAT1-1-1* fragment. We argue that an intramolecular recombination between these repeats occurs pre-meiotically in chromosomes of nuclei carrying the *MAT1-1;MAT1-2* locus, and results in a majority of nuclei with chromosomes carrying the typical *MAT1-1* locus. We propose that recognition between these two types of nuclei occurs prior to karyogamy, after which a typical meiosis produces self-fertile (*MAT1-1;MAT1-2*):self-sterile (*MAT1-1*) progeny in a ratio of 1:1. These structural discoveries and molecular manipulation allowed us to propose a mechanism whereby homothallic strains can yield both homothallic and heterothallic progeny.

## Results

### Molecular architecture of *MAT* loci in self-fertile and self-sterile strains of *C*. *spinulosa*

**Fig 1:**
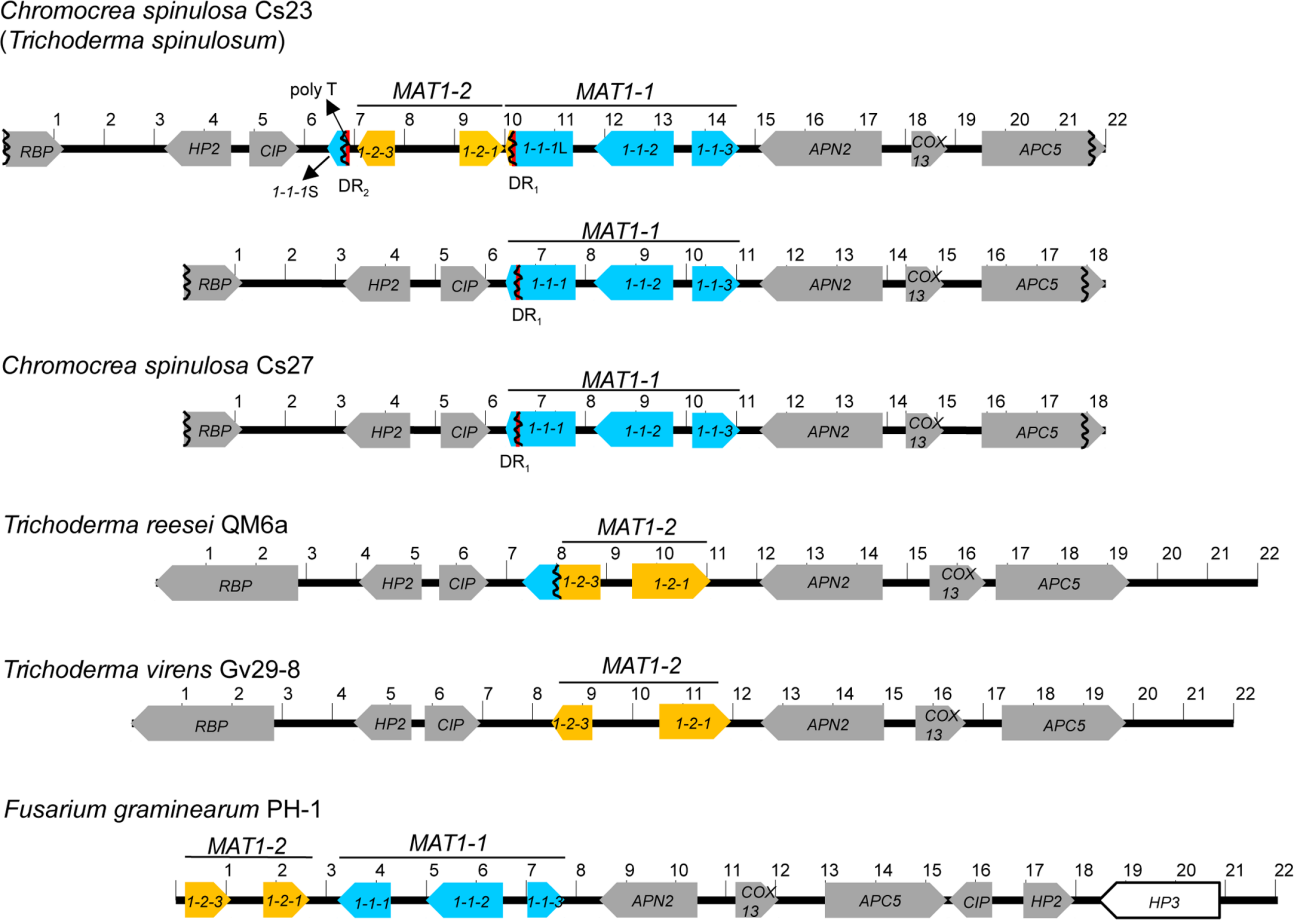
Structural organization of the *MAT* regions in homothallic and heterothallic *C*. *spinulosa* strains and comparisons with *MAT* in closely related heterothallic *Trichoderma* spp., and homothallic *F*. *graminearum*. Homothallic Cs23 has two *MAT* structures, while heterothallic Cs27 has one. Open reading frames (ORF) are depicted as large arrows indicating the direction of transcription. Homologs are indicated by the same gene name and color. Direct repeats (DRs) are shown as vertical red bars. *1-1-1*, *1-1-2*, *1-1-3*, *1-2-1*, *1-2-3*: *MAT1-1-1*, *MAT1-1-2*, *MAT1-1-3*, *MAT1-2-1*, *MAT1-2-3*, respectively. *MAT1-1-1* partial ORFs are indicated by a vertical wavy line. The *MAT1-1* genes (*MAT1-1-1*, *MAT1-1-2*, *MAT1-1-3*) and *MAT1-2* genes (*MAT1-2-1*, *MAT1-2-3*) are indicated by a horizontal bar. *HP*: encodes a hypothetical protein; *APN2*: similar to DNA lyase 2; *COX13*: similar to cytochrome c oxidase polypeptide VIa; *APC5*: similar to anaphase-promoting complex subunit 5; *CIP*: similar to complex I intermediate-associated protein 30; *RBP*: similar to a protein carrying RNA-binding motif. Note *MAT1-2-3* is fused to a fragment of *MAT1-1-1* in *T*. *reesii* QM6a. Numbers represent kilobases.

TAIL and inverse PCR-based chromosome walking strategies generated 21.9-kb and 18.3-kb contiguous DNA fragments from Cs23 and Cs27 strains respectively (Fig 1, S1 and S2 Figs, S1 and S2 Tables). Both Cs23 and Cs27 carry *MAT1-1* encoding the three canonical *MAT1-1* genes (*MAT1-1-1*, *MAT1-1-2*, and *MAT1-1-3*). Cs23 also carries a second version of *MAT* (*MAT1-1*;*MAT1-2*) with *MAT1-2* tightly linked to, and interrupting *MAT1-1-1* (described in detail below).

Six additional ORFs were identified in the *MAT* flanks and noted to be conserved and syntenic when compared to *MAT* flanks of closely related heterothallic *Trichoderma reesei* and *Trichoderma virens* (Fig 1). These genes are also conserved in homothallic *F*. *graminearum*, although with some rearrangement (Fig 1, bottom line). Interestingly, the *T*. *reesei* QM6a *MAT1-2* strain, which carries an intact *MAT1-2-1* ORF, contains a partial *MAT1-1-1* sequence (corresponding to 131 amino acids of the 3’ end) translationally fused to a hypothetical protein with similarity to *F*. *graminearum* MAT1-2-3 (28% over 55 amino acids out of 264 aa) [24] whose function is dispensable for self-fertility [25] (Fig 1). This *MAT1-1* locus structure is conserved among *MAT1-1* strains of *Hypocrea jecorina*, the teleomorphic stage of *T*. *reesei* [26]. In contrast, *MAT1-2-3* is present as a standalone ORF near *MAT* in *T*. *virens* strain Gv29-8 (protein ID 221797 JGI MycoCosm database (http://genome.jgi.doe.gov/TriviGv29_8_2/TriviGv29_8_2.home.html). Identification of two different architectures of *MAT1-2-3* in *Trichoderma* allowed us to speculate about *MAT* evolution in *C*. *spinulosa* self-fertile strain Cs23 (Discussion).

**Fig 2:**
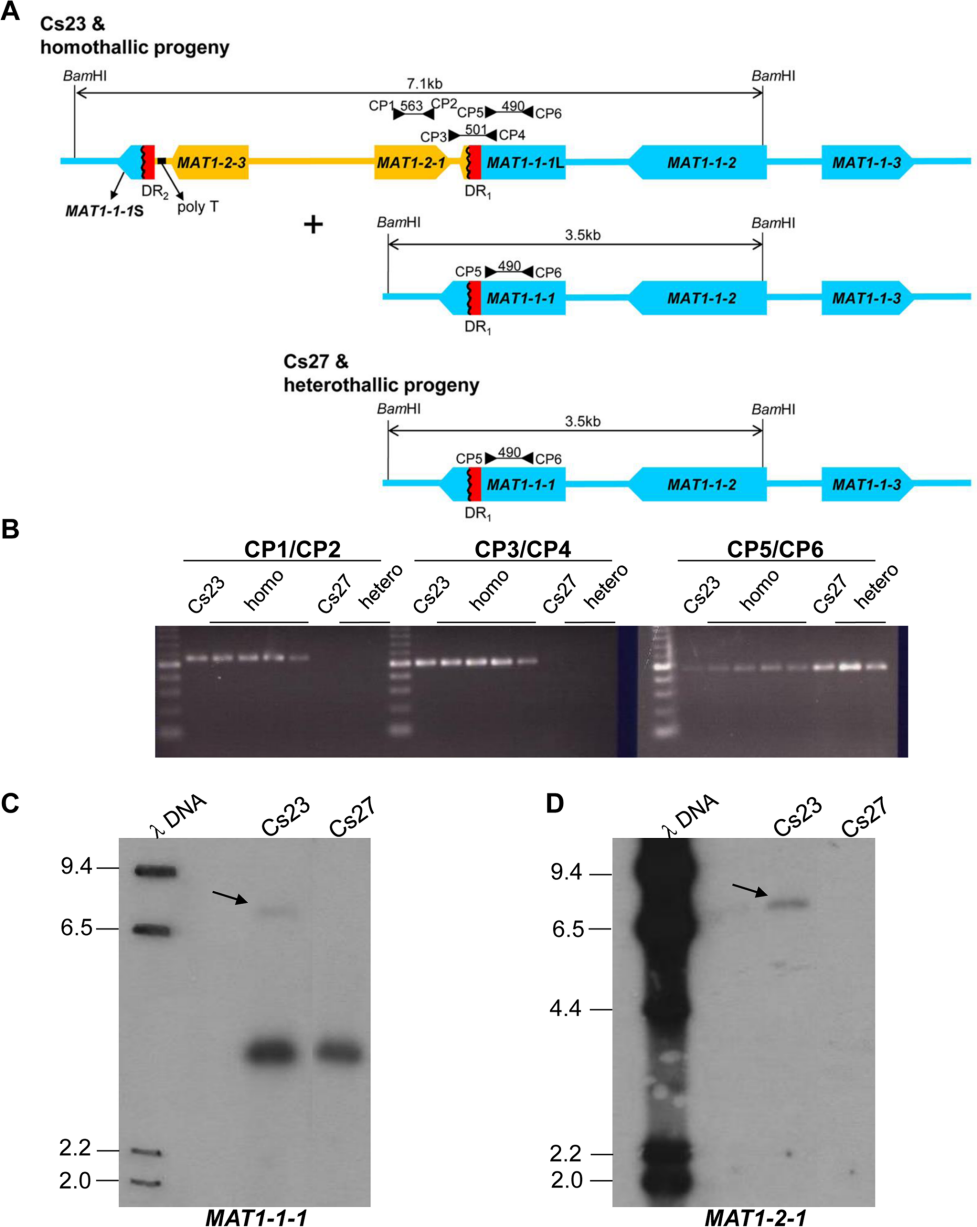
Structural organization of *MAT* in *C*. *spinulosa* homothallic and heterothallic strains. (A). Homothallic strains carry two versions of the *MAT* region, as shown. In the version with four *MAT* genes, two identical 115-bp direct repeats (designated DR_1_ and DR_2_) are present. DR_1_ is at the 3’ end of the larger truncated fragment of the *MAT1-1-1* ORF (*MAT1-1-1*L). DR_2_ is associated with the smaller truncated *MAT1-1-1* ORF (*MAT1-1-1*S) that is adjacent to *MAT1-2-3*. A homopolymeric tract (poly T, indicated by a black bar) is found in the region between *MAT1-1-1* and the *MAT1-2-3* ORF. In contrast, heterothallic strain Cs27 has only one (DR_1_) of the two direct repeats and no poly T tract. A second version of *MAT* with a structure similar to Cs27 *MAT* is also found in Cs23. *MAT1-1-1* is intact in this version. Primer positions, expected sizes of amplified fragments, and direction (arrowheads) are indicated. *Bam*HI sites and expected fragment sizes are shown.(B). PCR amplification of *MAT* genes from Cs23, Cs27, and progeny of a self of Cs23 using *MAT-*specific primers (indicated in A). Primer pairs CP1/2 and CP3/4 amplified products from homothallic strains only. Agarose gels: leftmost lanes in each panel: 100 bp ladder, homo: homothallic progeny, hetero: heterothallic progeny. (C-D). DNA gel blot of genomic DNAs from Cs23 and Cs27 hybridized with either *MAT1-1-1* (C) or *MAT1-2-1* (D) probes. λ DNA: lambda DNA digested with *Hin*dIII, Cs23 and Cs27: *Bam*HI-digested genomic DNA from *C*. *spinulosa* Cs23 and Cs27 respectively. The faint 7.1 kb band (C, arrows) was visible in DNA from Cs23 probed with either *MAT1-1-1* or *MAT1-2-1*. Cs27 hybridized (3.5 kb) to the *MAT1-1-1* probe but not the *MAT1-2-1* probe. The 3.5 kb fragment is also evident in Cs23, supporting the notion that there are two *MAT* structures in homothallic strains. The lane Cs23 (D) was from the same gel as the other lanes, but with intervening lanes removed. Sizes (in kb) are indicated to the left of the gel.

As noted above, two versions of Cs23 *MAT* are found; one version (*MAT1-1*) includes the three canonical *MAT1-1* genes while the second (*MAT1-1*;*MAT1-2*) includes four *MAT* genes, three of which, *MAT1-2-1*, *MAT1-1-2*, and *MAT1-1-3* are intact. The fourth, *MAT1-1-1*, is in two fragments, separated by ~3.5 kb of DNA that includes *MAT1-2-1*. The 5’ fragment of the *MAT1-1-1* ORF (*MAT1-1-1*L*)* is the largest (1140bp/380 amino acids) and contains the entire alpha 1 domain box. The 3’ fragment (*MAT1-1-1*S*)* is much smaller (76 aa). Two identical 115-bp DNA stretches (designated DR_1_ and DR_2_) reading in the same direction were identified. DR_1_ is 126 bp from the 3’ end of *MAT1-1-1*L (Fig 2). DR_2_ is at the 5’ end of *MAT1-1-1*S. Additionally, a homopolymeric tract (poly T), 155 bp from DR_2_ and an ORF encoding MAT1-2-3 are between *MAT1-1-1*S and *MAT1-2-1* (Figs 1 and 2). In contrast, in heterothallic strain Cs27, only one (DR_1_) of the two repeats is present at *MAT1-1*, *MAT1-1-1* is not fragmented, and no poly T tract or MAT1-2-3 protein are present (Figs 1 and 2).

### The *MAT1-1*;*MAT1-2* and *MAT1-1* loci are found in unequal proportions

Primer sets matching *MAT1-2-1* (CP1/CP2), *MAT1-1-1*L (CP5/CP6) and bridging DR_1_ (CP3/CP4) (S2 Table) amplified both *MAT1-2-1* and *MAT1-1-1* fragments and the DR_1_ region from DNA of C23 and self-fertile progeny, but only the *MAT1-1-1* fragment from DNA of C27 and self-sterile progeny (Fig 2A and 2B). In addition, diagnostic *MAT* fragments hybridized to DNA gel blots of self-fertile Cs23 and self-sterile Cs27 strains in a manner consistent with the PCR amplification pattern (Fig 2C and 2D). In Cs23, two fragments,7. 1 and 3.5 kb, corresponding to the *MAT1-1*;*MAT1-2* and *MAT1-1* versions of *MAT*, respectively, were visible when probed with *MAT1-1-1*, while only the 3.5 kb fragment was visible in Cs27 (Fig 2A, 2C and 2D). Notably, in the Cs23 lane, the intensity of the 7.1 kb signal (arrow) was much lower than that of the 3.5 kb signal (Fig 2C). The same gel, when probed with *MAT1-2-1*, hybridized to the 7.1 kb band in Cs23, also with low intensity (Fig 2D, arrow). The *MAT1-2-1* probe did not hybridize to Cs27. This is compelling evidence that the 7.1 kb band carries *MAT1-1-1* (*MAT1-1-1*L) and *MAT1-2-1* and that the copy number of *MAT1-1*;*MAT1-2* is less than that of *MAT1-1-1* on the 3.5 kb fragment. If the 3.5 kb *MAT1-1-1* and 7.1 kb *MAT1-1-1* and *MAT 1-2-1* signals were from DNA on the same chromosome in the same nucleus, or if Cs23 were heterokaryotic and the two types of nuclei were in equal numbers, the signals would be expected to be approximately the same intensity. We hypothesize that these unequal signals arise from DNA at the *MAT* locus and that the *MAT1-1*;*MAT1-2* locus and the *MAT1-1* locus reside in different nuclei that are unequally distributed in the population. To achieve this configuration, we propose that an intramolecular recombination occurs between the direct repeats in homothallic Cs23, eliminating *MAT1-2* and leaving an intact *MAT1-1-1* ORF as shown in S3 Fig. Note that the 3’ end of *MAT1-1-1*L extends 126 nucleotides beyond DR_1_, and this extra sequence would be eliminated in the loop out (S3 Fig). Following the loop out, the combined MAT1-1-1 protein aligns well with other MAT1-1-1 proteins particularly those from *Trichoderma* species. Whether or not the complete MAT1-1-1 protein is required for homothallic function or the *MAT1-1-1*L fragment is sufficient is addressed below.

### Does deletion of a direct repeat from the genome of Cs23 impact self-fertility?

**Fig 3:**
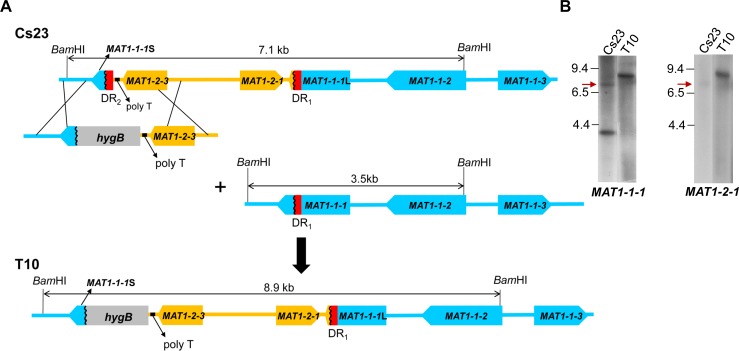
Targeted deletion of DR_2_ from Cs23. **(A).** A construct carrying the selectable marker *hygB* plus regions of homology flanking DR_2_ was introduced into WT self-fertile strain Cs23. Double crossover by homology would replace DR_2_ with *hygB*, otherwise the genomic architecture of the region is unchanged. T10, a strain with this type of replacement, also lacks the second version of *MAT* carrying only *MAT1-1*.**(B)** Gel blot of *Bam*HI-digested genomic DNA of Cs23 and candidate DR_2_-deletion transformant T10, hybridized with *MAT1-1-1* or *MAT1-2-1*. A band at 8.9 kb is evident in T10 with either probe and is of equivalent intensity. The original *Bam*HI-digested Cs23 *MAT1-2-1* fragment (7.1 kb) shown in (**A**) is indicated by red arrows. The 3.5 kb band is present only in Cs23 and only when probed with *MAT1-1-1*. Sizes (in kb) are indicated to the left of the gel. All lanes are from the same gel; irrelevant lanes between the lanes Cs23 and T10 were cut out of the image.

To determine if the direct repeats play a role in homothallic capability in Cs23, we deleted DR_2_ using the strategy shown in Fig 3A. DNA gel blot hybridization confirmed targeted replacement of the DR_2_ region with the *hygB* cassette via double crossover homologous recombination (Fig 3B). Specifically, *Bam*HI-digested genomic DNA of candidate deletion strain T10 showed a single 8.9-kb band hybridizing to both *MAT1-1-1* and *MAT1-2-1* (Fig 3B, lane 2) instead of the 7.1-kb band in progenitor Cs23 (Fig 3B, lane 1, arrow), confirming that DR_2_ had been replaced by *hygB*. Remarkably, the signal in T10 DNA hybridizing to either *MAT1-1-1* or *MAT1-2-1* was equally strong in contrast to the faint 7.1 kb signals in DNA of Cs23 (Fig 3B, arrows). Note also that the intense *MAT1-1-1* 3.5 kb signal in progenitor Cs23 is missing in T10 (Fig 3B), which suggests that T10 lacks the version of *MAT1-1* that is intact.

**Fig 4:**
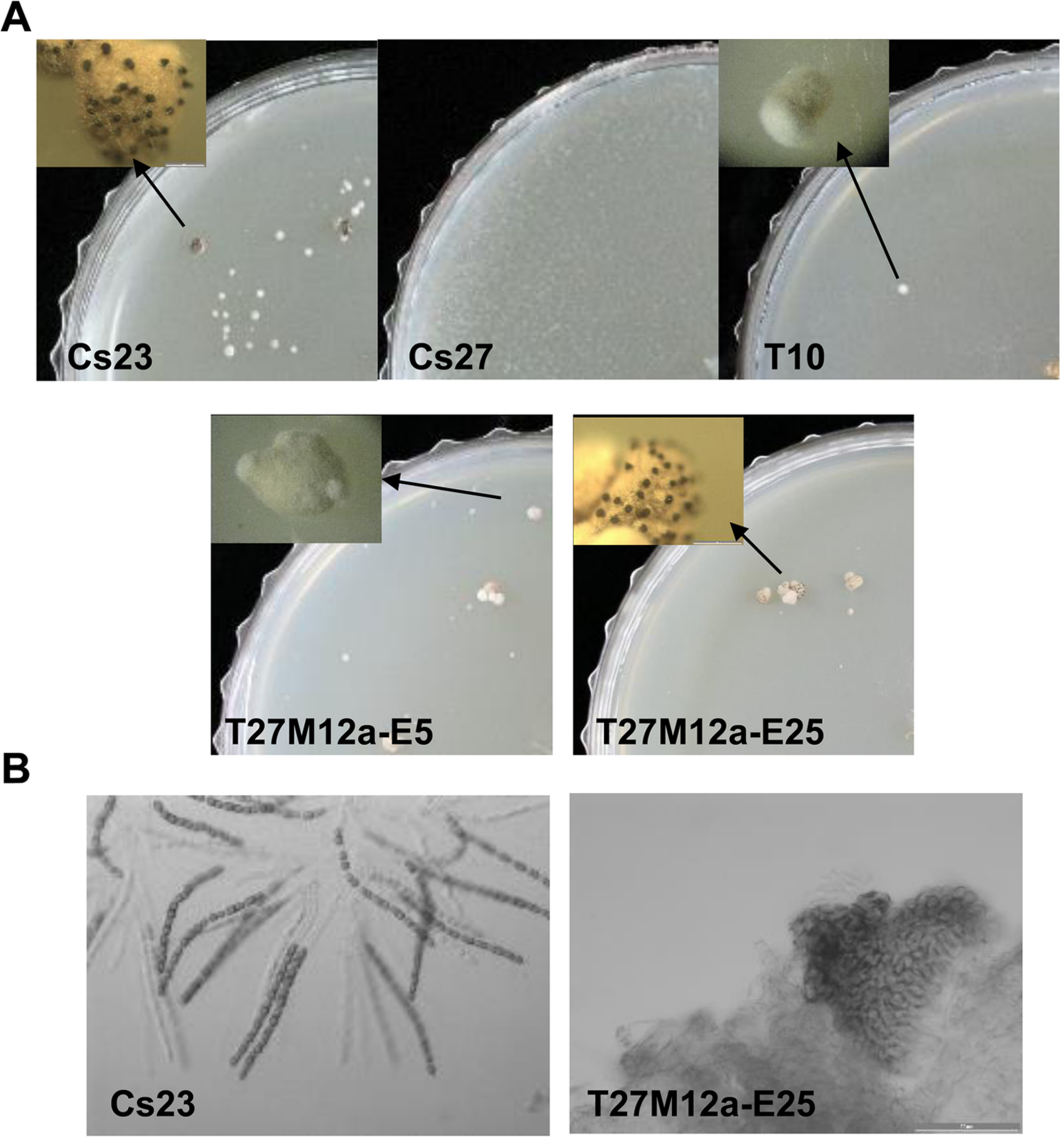
Self-fertility of WT and transgenic strains. **(A).** Self-fertile Cs23 forms stroma bearing perithecia (left panel inset, black dots at arrow) when grown on CMA, while self-sterile Cs27 does not. Strain T10 was able to form some stroma-like hyphal aggregates but no perithecia (top row inset, at arrow). Strains T27M12a-E5 and T27M12a-E25 formed stroma-like structures similar to those in Cs23, and, in the latter case, produced black perithecia immersed in the stroma, (but no asci/ascospores, B). **(B).** Self-fertile Cs23 forms stroma bearing perithecia with asci with 16 ascospores. Transgenic T27M12a-E25 forms stroma with perithecia but no ascospores.

The T10 strain maintained resistance to hygromycin B through 10 successive transfers on drug-free medium, indicating that it was mitotically stable. Unexpectedly, however, and in contrast to self-fertile Cs23, T10 was self-sterile on both PDA and corn meal agar (CMA) media [11], although it did form white, rounded hyphal aggregates (Fig 4A, top, insert). The T10 aggregates did not differentiate into stroma bearing sexual fruiting bodies (perithecia) as did the control Cs23 strain (contrast Fig 4A left and right, top row).

These findings suggest that the DRs are a key feature of the molecular mechanism generating the two versions of *MAT* in Cs23. Our demonstration that the DNA hybridization signals are unequal supports the notion that the two versions of *MAT* are in different nuclei.

### Can self-sterile Cs27 be made self-fertile by introduction of an intact copy of *MAT1-2*?

**Fig 5:**
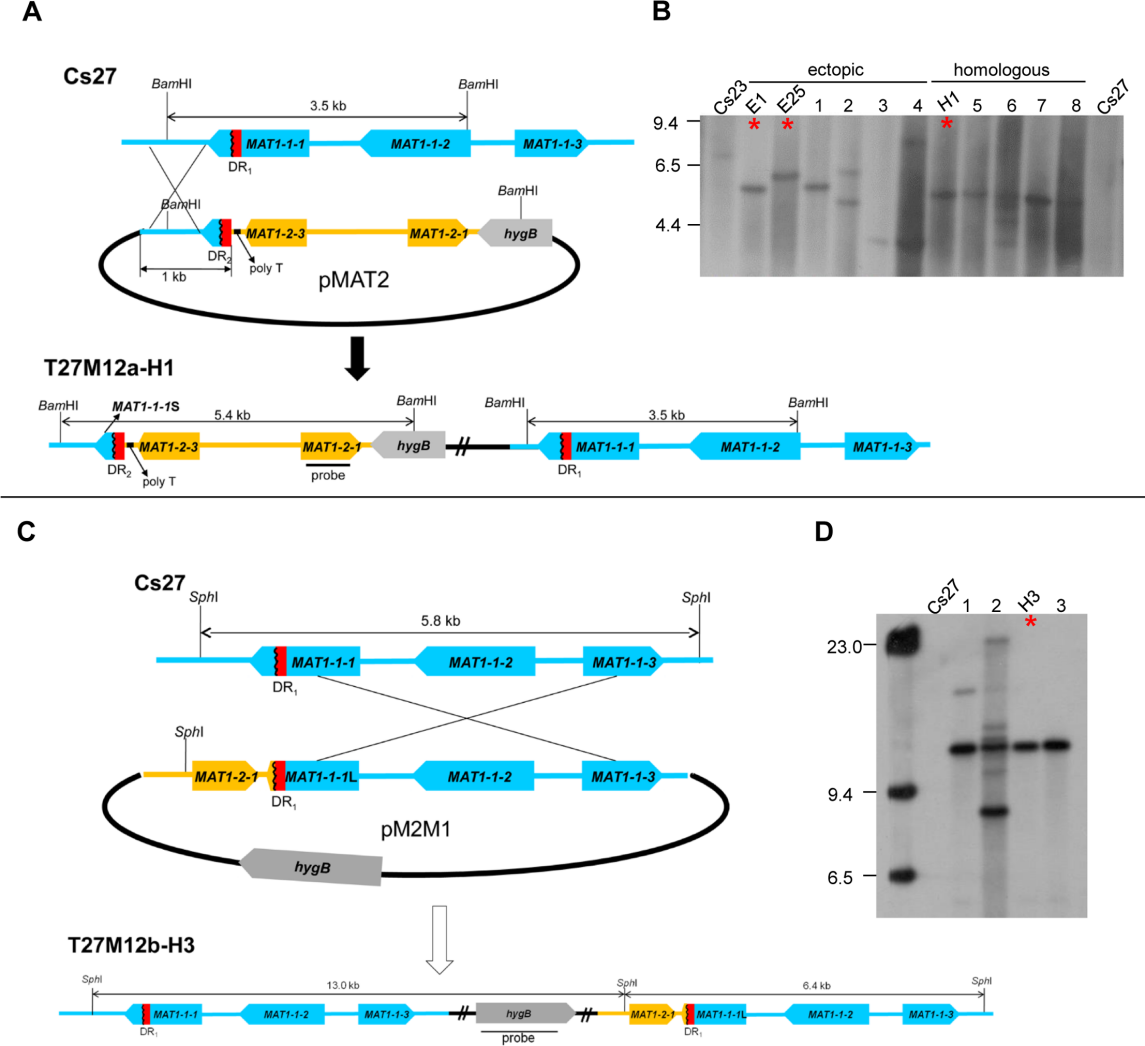
Introduction of the *MAT1-2* region from Cs23 into the genome of *C*. *spinulosa* Cs27. (**A and C**). Schemes to introduce *MAT1-2-1*. Plasmids pMAT2 and pM2M1 are described in the text. T27M12a-H1 (**B**, lane H1, asterisk) and T27M12b-H3 (**D**, lane H3, asterisk), represent homologous integrants from transformation with each type of plasmid. **(B)**. DNA gel blot of *Bam*HI-digested DNA from control strains Cs23 and Cs27, six transgenic strains (lanes E1, E25, 1–4) carrying *MAT1-2-1* at an ectopic position (ectopic), and five transgenic strains (lanes H1, 5–8) generated by homologous recombination as drawn in (**A**) (homologous), hybridized with a *MAT1-2-1* probe. Lane E1: T27M12a-E1; lane E25: T27M12a-E25; lane 1: T27M12a-E7; lane 2: T27M12a-E5; lane 3: T27M12a-E9; lane 4: T27M12a-E18; lane H1: T27M12a-H1; lane 5: T27M12a-H3; lane 6: T27M12a-H4; lane 7: T27M12a-H12; lane 8: T27M12a-H15. All homologous recombinants showed a single hybridizing band of expected size (5.4 kb). Asterisks highlight strains specifically described in the text. **(D)**. Gel blots of the *Sph*I-digested genomic DNAs from the control strain Cs27 and four transgenic strains carrying *MAT1-2-1*, hybridized with a *hygB* probe. Lane 1: T27M12b-H1; lane 2: T27M12b-H4; lane H3: T27M12b-H3; lane 3: T27M12b-H7. Integration by homologous recombination as drawn in (**C**) should generate a single hybridizing band of 13.0 kb (e.g., lane H3). Note some lanes had more complicated patterns likely reflecting both homologous and ectopic integration events. Sizes (in kb) are indicated on the left.

Two different Cs23 DNA fragments carrying *MAT1-2* were introduced into the genome of Cs27 which carries only *MAT1-1*. In the first case, a plasmid (pMAT2) bearing the *MAT1-2-1* ORF, truncated *MAT1-1-1*S/DR_2_/poly T/*MAT1-2-3* and 1.0 and 0.5-kb of the 5’ and 3’ sequences flanking these regions, respectively, was introduced (Fig 5A). In the second case, a plasmid (pM2M1) carrying all three *MAT1-1* genes (truncated *MAT1-1-1*L, *MAT1-1-2*, *MAT1-1-3*), as well as *MAT1-2-1*, was inserted (Fig 5C). Both homologous and ectopic integrants were obtained (Fig 5B and 5D). DNA gel blot analysis confirmed insertion of *MAT1-2-1* into the Cs27 genome (Fig 5B and 5D). Homologous integrants generated using each strategy are represented by T27M12a-H1 (Fig 5A, Fig 5B, lane 7, asterisk, Table 1) and T27M12b-H3 (Fig 5C, Fig 5D lane 4, asterisk, Table 1), respectively.

**Table 1:** Number of outcross progeny with each phenotype.

**Outcross 1**	**T10 (H**^**R**^**;SS) × Cs27 (SS)**			
	**Parental type**		**Recombinant type**	
	H^R^;SS	SS	SF	H^R^;SF
	(T10 type)	(Cs27 type)	(Cs23 type)	(Cs23 type)
	24	13	3	7
**Outcross 2**	T10-P48* **(H**^**R**^**;SS) × Cs27 (SS)**			
	**Parental type**		**Recombinant type**	
	H^R^;SS	SS	H^R^;SF	
	(T10 type)	(Cs27 type)	(Cs23 type)	
	24	42	4	
**Outcross 3**	T10-P102* **(H**^**R**^**;SS) × Cs27 (SS)**			
	**Parental type**		**Recombinant type**	
	H^R^;SS	SS	H^R^;SF	
	(T10 type)	(Cs27 type)	(Cs23 type)	
	15	44	4	
**Outcross 4**	T10-P48-4** **(H**^**R**^;SS) × TC27G-1*** **(G**^**R**^**;SS)**			
	**Parental type**		**Recombinant type**	
	H^R^;SS	G^R^;SS	G^R^;SF	H^R^;G^R^;SF
	(T10 type)	(Cs27 type)	(Cs23 type)	(Cs23 type)
	47	59	4	4

H^R^: resistant to hygromycin B, G^R^: resistant to geneticin, SS: self-sterile, SF: self-fertile

* T10-P48 and T10-P102 are T10-type (H^R^;SS) progeny obtained from outcross 1.

** T10-P48-4 is a T10-type (H^R^;SS) progeny from outcross 2.

*** TC27G-1 is a transgenic Cs27 strain carrying the *gen* marker in the 3'flank of *MAT1-1-3*.

Unlike T10 derived by deleting DR_2_ from Cs23, all transgenic strains generated by insertion of *MAT1-2-1* by homologous recombination into the *MAT* region of Cs27, were mitotically unstable. For example, *hygB*^*R*^ strain T27M12b-H3 (Fig 5C) completely lost resistance to hygromycin B after three successive transfers on PDA without the drug (S4 Fig). In the first transfer, these strains formed brownish stroma-like structures, similar in morphology to those formed by self-fertile Cs23, but no perithecia. In subsequent transfers, stroma-like structures no longer developed, although white hyphal aggregates, similar to those formed by T10 (Fig 4A top), were produced occasionally. T27M12a-H1 (Fig 5A) showed phenotypes similar to those of the T27M12b-H3 strain. In summary, all Cs27 transformants generated by homologous recombination, were mitotically unstable for *hygB* and did not produce normal-looking stroma (S4 Fig). We consider it likely that these are unstable because our introduced constructs created nearby repeated regions upon homologous integration.

In contrast, all of Cs27 transgenic strains examined (Fig 5B, lanes 1–4) carrying an intact copy of *MAT1-2-1* at an ectopic position (e.g. T27M12a-E5 in Fig 4A, Fig 5B, lane 1, asterisk) were mitotically stable, based on resistance to hygromycin B, and produced normal-looking stroma similar to stroma produced by self-fertile Cs23 (Fig 4A, bottom). However, none of these strains developed fertile perithecia on the stroma. One of these strains (T27M12a-E25, Fig 4, Fig 5B, lane 2, asterisk), produced black perithecia partially immersed in the stroma (Fig 4A, bottom), but no asci/ascospores (Fig 4B, bottom).

Thus, overall, all *hygB* stable transgenic *C*. *spinulosa* strains examined, whether from Cs23 or Cs27, were self-sterile. In the following, we focus on analyses of T10, the isogenic strain of Cs23 lacking DR_2_, and also on progeny from crosses in which T10 was a parent.

### Mating ability of strain T10 and progeny from outcrosses to Cs27

**Fig 6:**
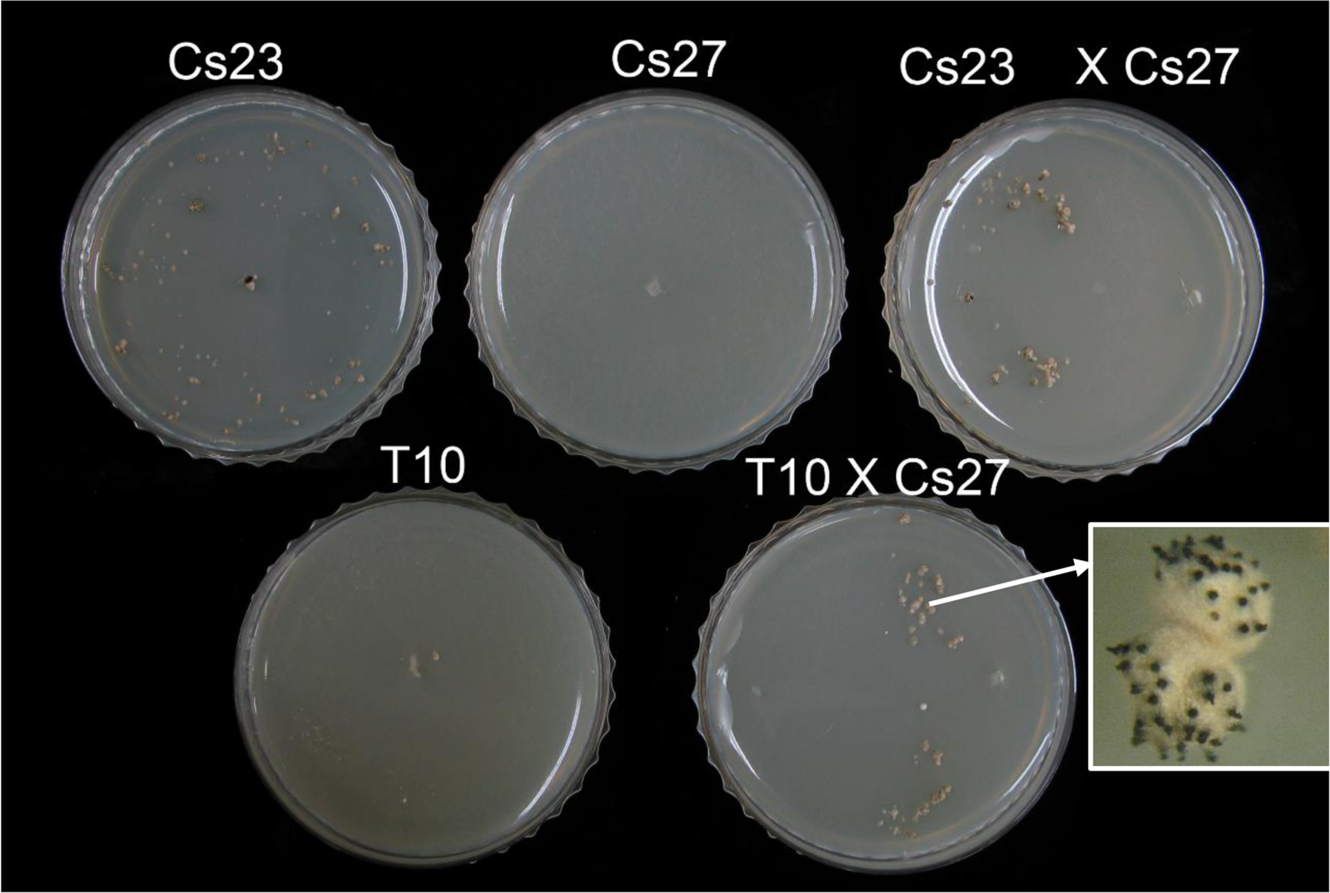
Outcrosses of self-sterile strain T10 to self-sterile Cs27 are fertile. Top row: Cross plates showing selfs of control Cs23 and Cs27 strains and a cross between them. Note stroma with perithecia formed on the Cs23 and the cross plate, as in Fig 4. Bottom row: Cross plates of a self of self-sterile T10 and a cross between T10 and self-sterile Cs27. Note formation of stroma bearing fertile perithecia on the cross plate.

Although strain T10 carrying both *MAT1-1* and *MAT1-2* was self-sterile, when out-crossed to self-sterile strain Cs27 carrying only *MAT1-1*, sexual progeny were produced (Fig 6). Four out-crosses were set up using the original T10 strain or T10-type progeny as one parent and Cs27 or geneticin-resistant (gen^R^) Cs27 strain TC27G-1 as the other parent (Table 1). The first cross was between the original T10 strain and Cs27, the second and third were between T10-type progeny obtained from the first out-cross, and Cs27, and the fourth was between T10-type progeny from the second out-cross and the genR Cs27 strain TC27G-1 carrying *gen* in the 3’ flank of *MAT1-1-3* (Table 1). As all self-sterile progeny were similar in both sexual development and colony morphology to the original parental T10 (hygB^R^, some hyphal aggregates) or Cs27 (hygB^S^, no stroma) strains, while the self-fertile progeny were identical to Cs23 (hygB^S^, perithecia), we designated these progeny T10-type, Cs27-type, and Cs23 type, respectively. The first out-cross produced progeny with phenotypes that were of four types. These included the parental types- hygB^R^ and self-sterile, like T10, and hygB^S^ and self-sterile, like Cs27, and two recombinant types- hygB^R^ and self-fertile and hygB^S^ and self-fertile. The fourth out-cross also produced progeny with four phenotypes. These included the parental types- hygB^R^ and self-sterile, like T10, and gen^R^ and self-sterile, like Cs27-G (Cs27), and two recombinant types- gen^R^ and self-fertile (Cs23 type) and hygB^R^gen^R^ and self-fertile (Cs23 type). The second and third crosses produced progeny with three phenotypes-hygB^R^ and self-sterile, like T10, hygB^S^ and self-sterile (like Cs27), hygB^R^ and self-fertile. Note that in all four crosses between self-sterile parents a low percentage of self-fertile progeny were recovered. No self-fertile progeny would be expected from a typical cross between self-sterile *heterothallic* parents.

The ratio of hygB^R^ to hygB^S^ progeny varied but was approximately 2:1, 1:2, and 1:2, in crosses 1–3, respectively. Self-fertile (Cs23 type) progeny were produced at frequencies of 21%, 5.7%, and 6.3% (Table 1). In the fourth out-cross, each parental phenotype (hygB^R^;self-sterile, and gen^R^;self-sterile) segregated approximately 1:1 (47:59), and recombinant phenotypes (hygB^R^;gen^R^;self-fertile and gen^R^;self-fertile) occurred with a frequency of 7%. In addition to the outcrosses described above, selfs of two hygB^R^ Cs23 type progeny obtained from outcross 1 produced both self-sterile (belonging to either the T10- or Cs27-type) and self-fertile progeny (Cs23 type). Self-fertile progeny were recovered at a much lower frequency (4/30 = 13.3%), than in selfs of the original Cs23 strain (~50% self-fertile and 50% self-sterile) in one cross only (Table 2). In addition, overall ascospore numbers were significantly lower than from selfs of the wild-type (WT) strain (~ 10% of Cs23).

**Table 2:** Phenotype of progeny from a self of the Cs23 type progeny derived from outcross 1 (Table 1).

**Self 1**	**P28 (H**^**R**^**;SF)**		
	H^R^;SS (T10 type)	SS (Cs27 type)	H^R^;SF (Cs23 type)
	17	9	4
**Self 2**	**P26 (H**^**R**^**:SF)**		
	H^R^;SS (T10 type)	SS (Cs27 type)	H^R^;SF (Cs23 type)
	5	7	0

H^R^: resistant to hygromycin B, SS: self-sterile, SF: self-fertile

Finally, unlike the T10-derived strains, none of the Cs27-derived transformants that carried *MAT1-2-1* at the *MAT1-1* locus or an ectopic position in the genome was able to produce fertile perithecia in outcrosses to Cs27. The genetic event(s) responsible for the occurrence of self-fertile progeny in any of the above outcrosses remains unclear.

### Molecular structure of *MAT* in progeny with different mating phenotypes

**Fig 7:**
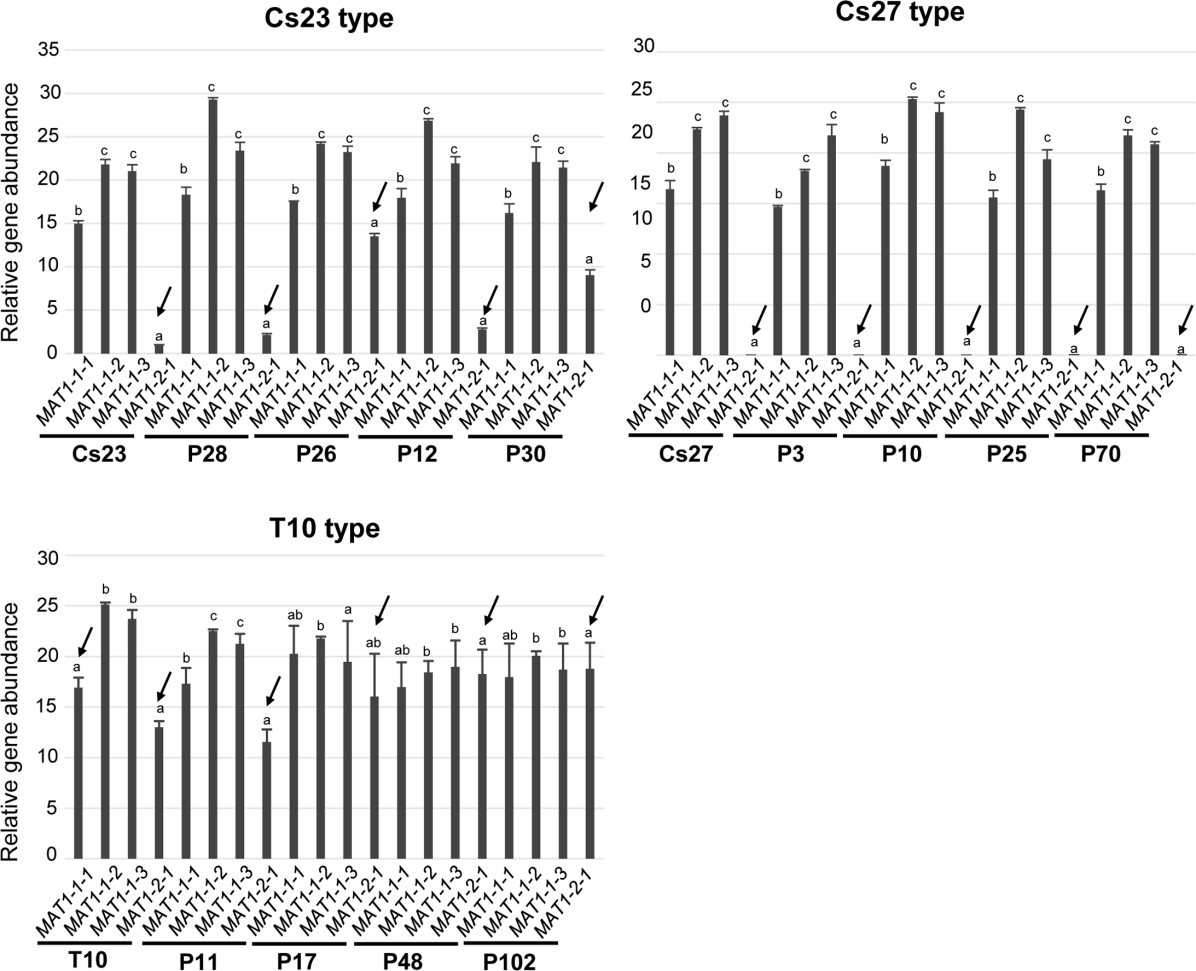
Relative amounts of individual *MAT* genes determined by qPCR using genomic DNA. Amplification level (amount) of *MAT1-2-1* is indicated by arrows, with *MAT1-2-1* level in Cs23 as a reference. The same letter above bars indicates no significant difference. The three panels represent Cs23 and Cs23-type progeny, C27 and Cs27-type progeny and T10 and T10-type progeny from the T10 by Cs27 crosses. Primer pairs used: for MAT1-1-1: qCsmat1-1for7 & qCsmat1-1rev7, for MAT1-1-2: qCsmat1-2for6 & qCsmat1-2rev6, for MAT1-1-3: qCsmat1-3for4 & qCsmat1-3rev4, for MAT1-2-1: qCsmat2for4 & qCsmat2rev4.

**Fig 8:**
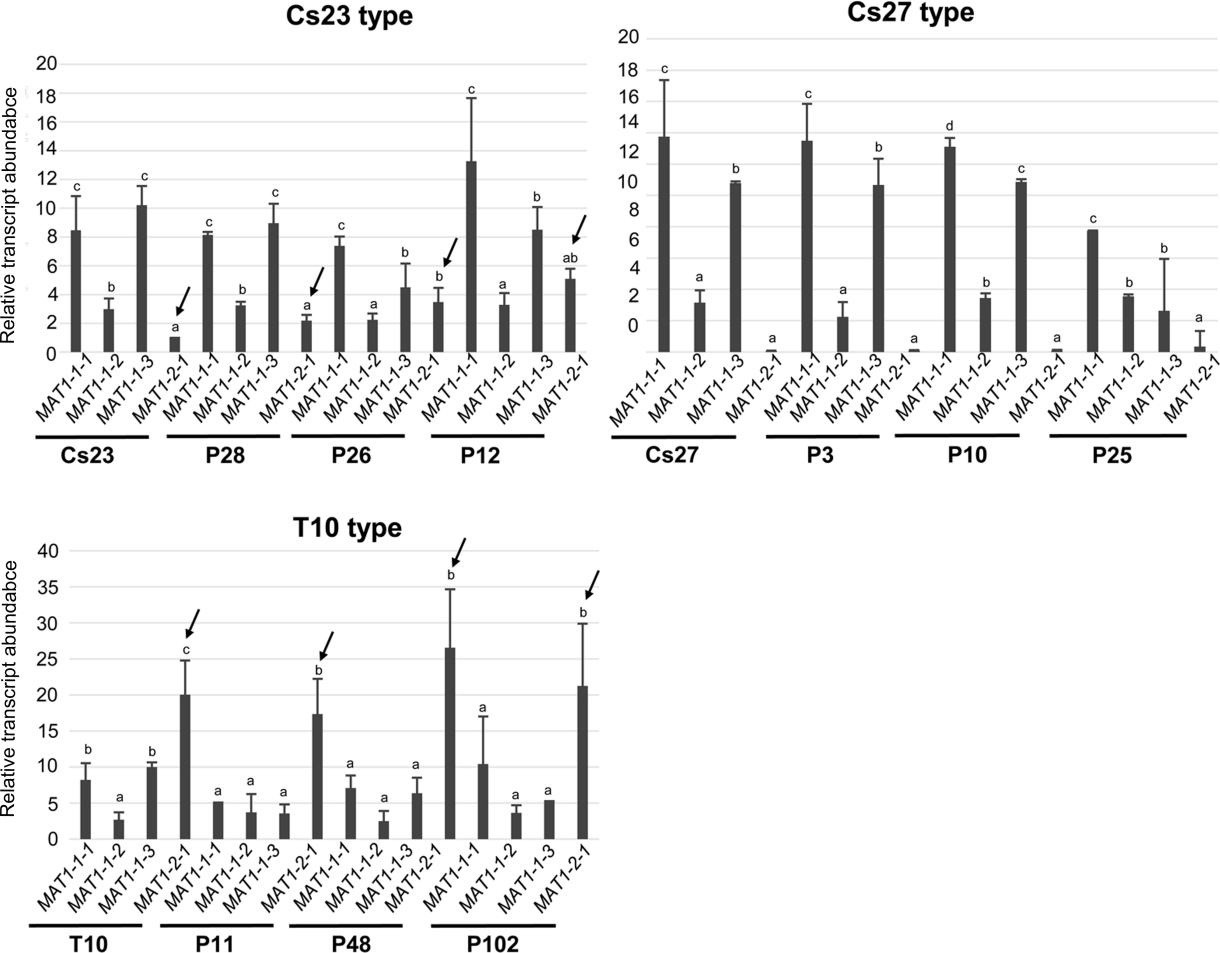
Relative transcript levels for individual *MAT* genes determined by qPCR using total RNA. Amplification level (amount) of *MAT1-2-1* is indicated by arrows, with *MAT1-2-1* level in Cs23 as a reference (set to 1). The same letter above bars indicates no significant difference. The three panels represent Cs23 and Cs23-type progeny, C27 and Cs27-type progeny and T10 and T10-type progeny from the T10 by Cs27 crosses, as in Fig 7. Primer pairs used as in Fig 7.

We analyzed the structure of *MAT* loci in progeny of out-crosses described above, using DNA gel blots (S5 Fig) and quantitative real-time PCR (qPCR) (Figs 7 and 8). The appearance of a single hybridizing 2.6 kb-*Sac*I-digested band (‘**b**’ in S5 Fig) in the DNA of T10-type progeny, probed with *MAT1-1-1* or a DR region, demonstrated that all T10-type progeny examined carried *MAT1-1-1*L and one DR near *MAT1-1-1*L as in the original T10 strain (S5A–S5C Fig). Additionally, the 6.1 kb-*MAT1-2-1-*hybridizing band (‘**a**’ in S5D Fig) showed intensities in T10-type progeny similar to those hybridizing to *MAT1-1-1*L (S4A and S4C Fig), supporting the genomic architecture of the *MAT* loci depicted in S5A Fig (top panel). Note that the 2.6 and 6.1 kb bands were not visible in progenitor Cs23 DNA, again supporting the notion that nuclei containing *MAT1-2-1* are in the minority in this strain. The 3.3-kb hybridizing band (‘**c**’ in S5B and S5C Fig) confirmed the presence of only *MAT1-1* in Cs27-type progeny, as in the original Cs27 strain. This band is also visible in Cs23 as expected.

In contrast, in some Cs23-type self-fertile progeny (P12, P26, P30, and P60), two *Sac*I bands (3.3 and 2.6 kb) were visible (S5B and S5C Fig) demonstrating that these progeny carried both versions of *MAT* (S5A Fig, middle). The ratio of intensities of the two hybridizing bands varied, suggestive of unequal numbers of nuclei carrying each *MAT* structure, as proposed for the original Cs23 strain (Fig 2).

We performed qPCR on genomic DNA to determine copy number of individual *MAT* genes in the progeny that we examined by Southern hybridization (Fig 7). In Cs23 and Cs23-type progeny, the relative amounts of *MAT1-1-2* and *MAT1-1-3* did not differ significantly from each other in any of the strains examined. *MAT1-1-1* was less abundant than these two *MAT1-1* genes, but the largest fold-changes among the three *MAT1-1* genes in the same strain were less than ~1.7 (e.g., Cs23 type progeny P28 and P26, Fig 7). In contrast, they were ~15- to ~25-fold-higher than the relative amount (1.0) of *MAT1-2-1* in several Cs23 types (e.g., Cs23, P12 and P28). The relative amounts of *MAT1-2-1* in T10 and T10-type progeny did not differ significantly (less than a two-fold change) from those of the *MAT1-1* genes in the same strain. In particular, T10 and progeny P17, P48, and P102 showed *MAT1-2-1* levels similar to those of the *MAT1-1* genes within the same strain (e.g., ~0.8- to ~1.1-fold changes compared with *MAT1-1-3*). This is in marked contrast to the *MAT1-2-1* levels in Cs23 type progeny which were much lower than those of the *MAT1-1* genes, ranging from ~0.04- to ~0.4-fold compared with *MAT1-2-1* in the same progeny. No *MAT1-2-1* signal was detected in Cs27 and Cs27 type progeny and the profiles of all progeny mirrored those of Cs27 (Fig 7).

### Expression of *MAT* genes in parents and progeny

We examined expression patterns of each *MAT* gene in a subset of the progeny shown in Fig 7 and S5 Fig by qPCR using total RNAs from progeny grown on PDA (Fig 8). In all strains examined, the *MAT1-1-2* transcript accumulated at the lowest levels among the three *MAT1-1* transcripts at the *MAT1-1* locus, which is consistent with the expression pattern of the three *MAT1-1* transcripts in self-fertile *F*. *graminearum* [25]. The levels of the *MAT1-2-1* transcript varied among the strains examined. In Cs23 and Cs23-type progeny the signal was lower than those of the *MAT1-1* genes (lower than those of all three *MAT1-1* transcripts in Cs23 and P28 strains, and lower than those of the *MAT1-1-1* and *MAT1-1-3* transcripts in P12 and P26 strains). In contrast, *MAT1-2-1* transcripts in the T10-type progeny showed the highest accumulation levels among all *MAT* transcripts, ranging from ~4- to ~13-fold higher than *MAT1-1-2* transcripts. The expression pattern of *MAT1-2-1* in the T10-type progeny was consistent with that of *MAT1-2-1* in *F*. *graminearum*, where the transcript level of *MAT1-2-1* was the highest of all *MAT* genes [25]. In contrast, no significant *MAT1-2-1* expression was detected in Cs27 and Cs27-type strains.

To determine whether or not *MAT1-1-1*L was expressed, we used qPCR analysis of total RNA with PCR primers (S6 Fig, S2 Table) that could distinguish *MAT1-1-1*L from intact *MAT1-1-1* (S7 Fig). These experiments revealed that the *MAT1-1-1*L fragment was indeed expressed in Cs23 and Cs23-type progeny (*MAT1-2-1* was set to as reference) because the expression level of *MAT1-1-1*L was almost same as that of *MAT1-2-1*. In contrast, *MAT1-1-1*L transcript levels in T10 or T10-type progeny were much higher than those in Cs23 strains (S7A Fig).

qPCR on genomic DNA determined that copy number of *MAT1-1-1*L was less than *MAT1-1-1*. *MAT1-1-1*L signals from genomic DNA were not significantly different from those of *MAT1-2-1* (set to 1 as reference) in Cs23 and Cs23-type progeny, whereas *MAT1-1-1*L signals in T10 strains were higher but also similar to those of *MAT1-2-1* (S7B Fig).

### Test of MAT1-1-1L protein function by heterologous expression in a *MAT1-1-1* deletion strain of *F*. *graminearum*

To determine if Cs23 *MAT1-1-1*L is functional, we inserted it and *MAT1-1-1* (as a positive control), separately, into a *F*. *graminearum* Δ*MAT1-1-1* strain [25] (S8A Fig). PCR amplification analysis revealed that all candidate hygB^R^, gen^R^ strains, transformed with *C*. *spinulosa MAT1-1-1* (designated FgΔ*MAT1-1-1*::Cs*MAT1-1-1*), *MAT1-1-1*L (FgΔ*MAT1-1-1*::Cs*MAT1-1-1*L) or *F*. *graminearum MAT1-1-1* carried the transgenes at ectopic positions (S8A Fig). Wild-type self-fertile *F*. *graminearum* positive control strain Z3643 and all transgenic (FgΔ*MAT1-1-1*::Fg*MAT1-1-1* strains, began to form protoperithecia at 3 dai (days after inoculation) and were fully fertile after 6–7 dai, containing asci with eight ascospores. The negative control strain, *F*. *graminearum* Δ*MAT1-1-1* formed perithecia that were 4~5 times smaller than WT perithecia and barren even four weeks after perithecial induction (S8B and S8C Fig). Transgenic FgΔ*MAT1-1-1*::Cs*MAT1-1-1* and FgΔ*MAT1-1-1*::Cs*MAT1-1-1*L strains also produced small, barren perithecia, but these were at least two times bigger than those of the negative control, and were not significantly different from each other (S8B and S8C Fig). Although they could not completely complement function, both *C*. *spinulosa MAT1-1-1*L and *MAT1-1-1* performed similarly, thus we conclude that both are able of promoting perithecial development in *F*. *graminearum* and that *MAT1-1-1*L is functional.

### Staining of ascospore nuclei with DAPI

We attempted to determine whether nuclei in large versus small spores could be distinguished using DAPI staining. Ascospores generated by selfing Cs23 revealed that only immature asci in which ascospores could not be seen clearly or were just starting to be visible were stained well; mature tetrads (carrying 16 ascospores of two sizes) were barely stained (S9A and S9B Fig). Immature asci carrying nuclei in various stages of division (ranging from nuclei in the diploid zygote to those in complete tetrads) were distinguishable (S9A andS9B Fig). The presence of several types of ascospore arrangement within an ascus (8L:8S, 4L:8S:4L, 4S:8L:4S, 4L:4S:4L:4S; large = L, small = S) indicates that second, as well as first meiotic division segregations occurred frequently. At least 2 to 3 nuclei were present per single ascospore, though the exact number was unclear (S10 Fig). As they matured, nuclei in small ascospores were less likely to be stained than in large (S10 C, D, leftmost ascus), which may suggest that small ascospores mature earlier than large ones. Nevertheless, no significant difference between large and small ascospores within as ascus (number/or intensity of nuclei) was found.

Asci from an outcross between T10 and Cs27 were not significantly different from those from the Cs23 self in terms of spore number and size segregation (S9C and S9D Fig). However, the frequency of asci segregating for size in the first meiotic division was different between the Cs23 self (22.9%, 8 out of 35 asci) and the outcross (60.5%, 17 out of 25 asci).

## Discussion

**Fig 9:**
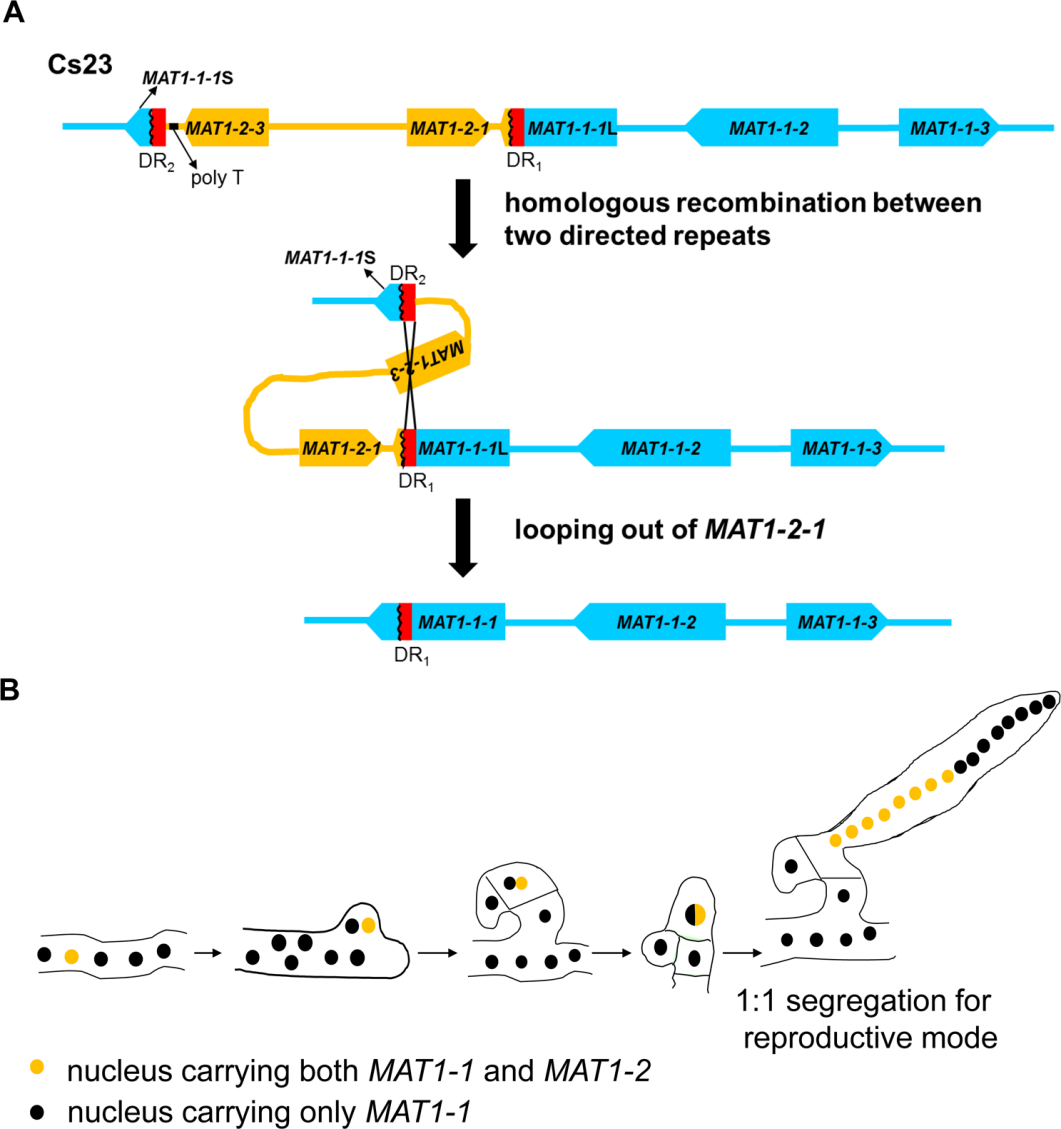
Proposed model for elimination of *MAT1-2* and acquisition of concomitant homothallic capability involving recognition between two types of nuclei in the same individual. **(A).** Looping out of the *MAT1-2* region from self-fertile *C*. *spinulosa* strain 23 by homologous recombination between the 115-bp direct repeats. **(B).** Model for *C*. *spinulosa* ascus development. The two types of nuclei in the ascogenous hyphae are present in unequal proportions, with nuclei carrying *MAT1-1*;*MAT1-2* in the minority. A nucleus carrying *MAT1-1*;*MAT1-2* (yellow) recognizes and pairs with a nucleus carrying *MAT1-1* only (black) and migrates into the developing crozier. The nuclear pair are partitioned in the penultimate cell of the crozier where they undergo karyogamy, meiosis, and ascus development. The diploid nucleus undergoes meiosis followed by two rounds of mitosis, yielding 16 ascospores segregating 1:1 ratio for self-fertility and large size *versus* self-sterility and small size in a single ascus.

As described above, WT self-fertile strain Cs23 (Figs 1 and 2, S1 Fig) carries two versions of *MAT*, one with *MAT1-2* closely linked to *MAT1-1* (*MAT1-1*;*MAT1-2*), the other with only *MAT1-1*. The architecture of the former is similar to *MAT* structure in several other homothallic Sordariomycetes, including *F*. *graminearum*, in that it includes genes encoded at both *MAT1-1* and *MAT1-2* counterparts of heterothallic species [27]. Cs23 *MAT1-1*;*MAT1-2*, however, has several noteworthy structural variations. Direct repeats are found 5’ and 3’ of *MAT1-2-1*, the *MAT1-1-1* ORF is interrupted by *MAT1-2-1* and both fragments of *MAT1-1-1* are bordered by a repeat. In contrast, the WT self-sterile strain Cs27 (S10 Fig) carries only *MAT1-1* with three complete genes (Figs 2 and 9). As noted above, this form of *MAT* is also found in homothallic Cs23.

Development of several different types of *MAT*-manipulated strain provided insight into mechanisms of homothallism and unidirectional mating-type alteration in *C*. *spinulosa*. One type of transgenic strain, derived from Cs23, carried the *MAT1-1*;*MAT1-2* organization but lacked one of the DRs. The poly T tract and *MAT1-2-3* were intact. These manipulations provide robust evidence that the DRs, but not the poly T tract, or the MAT1-2-3 protein are key to homothallic behavior. Transgenic strains generated from Cs27, carried a copy of *MAT1-2-1* introduced adjacent to the *MAT1-1* locus by homologous recombination or integrated at an ectopic position. These strains have the two repeats and an intact *MAT1-2* and *MAT1-1*. One version typified by homologous integrant T27M12a-H1 (Fig 5) also carried the poly T tract and *MAT1-2-3*, while the other version, typified by homologous integrant T27M12b-H3 lacked poly T and *MAT1-2-3* and had a duplication of most of the *MAT1-1* region (Fig 5). We consider it likely that the mitotic instability we detected for both types of transgenic strain reflects an inherent instability trait of the *MAT* region that is tied to the mechanism of homothallism. Naturally occurring Cs23 is mitotically unstable due to naturally occurring repeated regions, while our engineered Cs27 strains are mitotically unstable because we introduced repeated regions at *MAT*. This notion is supported by the fact that Cs27 transgenic strains carrying an ectopic copy of *MAT1-2* were mitotically stable. Mating assays with these strains, along with qPCR of *MAT* DNA and RNA, provided data crucial for addressing the following questions.

### Are the direct repeats associated with unidirectional mating-type alteration in *C*. *spinulosa?*

The presence of 115-bp direct repeats bordering the *MAT1-2* locus in Cs23 suggested a possible mechanism for elimination of *MAT1-2-1*, based on homologous recombination between the DRs, as pointed out by us in preliminary work [22]. Such an event would loop out *MAT1-2-1*, leaving only the *MAT1-1* locus, now with an *intact MAT1-1-1* ORF. Importantly, this would result in two types of nuclei in a common cytoplasm, one type carrying *MAT1-1*;*MAT1-2* and the other type carrying *MAT1-1* only, as is actually found in homothallic Cs23 (Fig 9).

The weak intensity of the band hybridizing to *MAT1-2-1* compared with the band hybridizing to *MAT1-1-1* on DNA gel blots of Cs23 (Fig 2) supports a model in which a minority of nuclei carry both *MAT* versions during mitotic growth (Fig 9B, S10 Fig). That the DRs are important in elimination of *MAT1-2-1* and subsequent unevenness of nuclei carrying this *MAT* gene was confirmed by comparing DNA blots of WT Cs23 to those of transgenic strains derived from it (T10 and T10-type progeny) that lack DR_1_ and are self-sterile. Unlike Cs23, which shows two hybridizing bands when hybridized with *MAT1-1-1*, one of which also hybridizes to *MAT1-2-1*, T10 strains showed only one, and the same, band, when hybridized separately with *MAT1-1-1* and *MAT1-2-1* (Fig 3). Furthermore, in contrast to WT Cs23, the intensities of bands hybridizing to *MAT1-1-1* or *MAT1-2-1* in T10 were similar (Fig 3, S4 Fig). This indicates that the *MAT1-1*;*MAT1-2* organization in T10 was stably maintained during vegetative growth and sexual development. T10 strains were no longer self-fertile, which bolsters our hypothesis that removal of one repeat from Cs23 would prevent looping out of *MAT1-2-1*, and abolish capability for unidirectional mating-type alteration and self-mating.

Although *MAT1-1-1* at the *MAT1-1*;*MAT1-2* locus is split to a large (*MAT1-1-1*L) and small (*MAT1-1-1*S) fragment, this may not lead to functional disruption. *MAT1-1-1*L, which consists of ~83% of *MAT1-1-1* including the alpha-box (S3 Fig), is expressed (S7 Fig) and capable of promoting perithecium formation as well as the intact Cs*MAT1-1-1* in a *MAT1-1-1*-deletion strain of *F*. *graminearum* (S8 Fig). Therefore, it is likely that Cs23 carries two different types of nuclei, one expressing three functional *MAT1-1* genes (including *MAT1-1-1*L) and *MAT1-2-1*, and the other expressing only *MAT1-1* genes (including *MAT1-1-1*), and that karyogamy between these two different nuclei is necessary for sexual development (see details below).

To date, the involvement of DNA rearrangement in mating-type switching has been elucidated in depth for only a few yeast species that show bi-directional switching. *S*. *cerevisiae*, which carries three *MAT* loci (an active *MAT* locus and two silent *MAT* loci) on the same chromosome, can switch mating type through a mitotic recombination-dependent gene conversion between the silent and active copies [7]. The heavily studied yeast model, *S*. *pombe* operates similarly [28]. Beyond the Saccharomycetaceae with three *MAT* loci [29,30], methylotropic yeasts such as *H*. *polymorpha* and *P*. *pastoris*, which carry two linked *MAT* loci, undergo mating-type inversion mediated by inverted repeats [8,9], that silences one of the two *MAT* loci and thus changes mating type [10]. Our in depth probing of *MAT* alteration with the filamentous Sordariomycete *C*. *spinulosa* supports our earlier hypotheses [22] and has parallels with recently described features of mating behavior of the Leotiomycete, *S*. *trifoliorum* [21] and the Sordariomycete, *C*. *fimbriata* [20] Although the gene complement at *MAT* in homothallic strains of these species varies, both carry *MAT1-1* and *MAT1-2*, and have structural features (two direct repeats flanking *MAT1-2*) that testify to a mechanism by which the *MAT1-2* region is eliminated via homologous recombination between repeats (Fig 9A). S3 Table summarizes the similarities and differences among *C*. *spinulosa*, *S*. *trifoliorum*, and *C*. *fimbriata MAT* features. To our knowledge no functional analyses by gene manipulation have been reported for *S*. *trifoliorum* or *C*. *fimbriata*. Through DR and *MAT-*manipulation, crossing experiments with transgenic strains, and examination of *MAT* gene copy number and expression levels in WT strains and progeny, we provide experimental evidence for the looping mechanism and demonstrate that the *MAT1-1*;*MAT1-2* region with associated DRs is unstable during vegetative growth. This feature is central to the mechanism of homothallism. *Crucially*, *this process also leaves two types of nuclei*, *in unequal numbers*, *in a common cytoplasm*.

### Is maintenance of opposite mating-type loci in a single nucleus sufficient for homothallism in *C*. *spinulosa?*

An unexpected result of this study was that transgenic *C*. *spinulosa* strains carrying both *MAT1-1* and *MAT1-2* loci in a single nucleus were self-sterile. Transgenic T10 and T10-type progeny, with *MAT1-1*;*MAT1-2* architecture identical to that of Cs23 except for the absence of DR_1_ (replaced with hygB) were unable even to produce stroma, although all four *MAT* genes were transcribed to levels similar to levels in self-fertile Cs23 on PDA medium. Transgenic Cs27 strains carrying an intact *MAT1-2-1* gene at an ectopic position produced normal-looking stroma with occasional perithecia, however all were barren. These results strongly suggest that presence of opposite forms of *MAT* in a single nucleus is not sufficient for self-fertility in *C*. *spinulosa*, even though this appears to be sufficient for many fungi that self successfully [22,27,31,32]. Supporting our findings are several reports that *heterothallic* species carrying both the native *MAT* locus and an introduced *MAT* locus of opposite mating type are not fertile [33–36].

### What is the molecular mechanism for homothallism during unidirectional mating-type switching in *C*. *spinulosa?*

Based on the unequal intensity of *MAT1-1-1*- and *MAT1-2-1*-hybridizing bands on DNA gel blots of Cs23, we suggest that Cs23 hyphae carry two different types of nuclei in unequal numbers. One type contains the *MAT1-1*;*MAT1-2* structure and the other, *MAT1-1* only (Fig 9, S10 Fig). We propose that this unusual *MAT* organization is achieved by deletion of *MAT1-2-1* from a majority of the nuclei within a common cytoplasm during mitotic growth, via a DR-mediated looping out mechanism that reconstructs the full *MAT1-1-1* ORF and leaves the *MAT1-1* locus only. Because the *MAT1-1-1*L fragment is expressed and appears to be as functional as the intact *MAT1-1-1* ORF in a *MAT1-1-1*-deletion strain of *F*. *graminearum*, we hypothesize that this loop out step is required for subsequent karyogamy. Given that T10-type strains, constructed in the Cs23 genetic background, but carrying only one version of *MAT* with both *MAT1-1* and *MAT1-2*, were completely self-sterile, we propose that karyogamy in WT Cs23 occurs *only* between the two types of nuclei during sexual development (Fig 9B). Subsequent meiosis would result in production of progeny segregating 1:1 for parental type nuclei (Fig 9B). Specifically, 50% of these progeny would carry both *MAT* loci and be self-fertile and large [11], while the other 50% would carry only *MAT1-1* and be self-sterile and small. When self-fertile progeny are selfed, the same genetic event (i.e., the loss of *MAT1-2-1* during mitotic growth) is repeated.

It remains unclear, and a subject of further study, how the two types of nuclei recognize each other. Do they behave as functionally heterothallic, perhaps by a mechanism that allows for activation of *MAT1-2-1* expression and repression of expression of the three *MAT1-1* genes at a critical stage in nuclei with *MAT1-1*;*MAT1-2*? Differential expression and epigenetic inactivation of *MAT* have been hypothesized [1,2,37]. Also unknown are the particulars of the molecular mechanism underlying the DR-mediated homologous recombination in *C*. *spinulosa*. *MAT1-2-1* deletion occurs at high frequency in Cs23 during mitotic growth (most nuclei do not carry it, Fig 9, S10 Fig). Given that DR_1_ and DR_2_ are relatively short (115-bp) compared with in the size of inverted repeats (> 2 kb) in methylotropic yeasts [8,9], it is likely that additional genetic element(s) (e.g., site-specific recombinases or transposases) are required for enhancing this recombination event.

In addition, it is difficult at this point, to explain the low frequency of self-fertile progeny in out-crosses between T10 (or T10-type progeny) and Cs27 (Table 1). When selfed (Table 2), one of these self-fertile progeny (P28) produced both self-fertile and self-sterile progeny, but the other (P26) did not. One clue might be that the copy number of *MAT1-2-1* in P28 matched that of Cs23, while in P26, *MAT1-2-1* copy number was higher (Table 2, Fig 7). Note, however, that the ratio of self-sterile to self-fertile progeny in the P28 self was not 1:1 as in self-fertile WT control Cs23 (Table 2). The connection between ascospore size and *MAT* genotype remains a mystery.

### How does the *MAT* chromosome evolve in *C*. *spinulosa?*

Although the molecular mechanism underpinning the evolutionary origin of *C*. *spinulosa* DRs located at the Cs23 *MAT* locus remains unclear, the availability of *MAT* sequences of several closely related *Trichoderma* species allows us to propose a mechanism for evolution of *C*. *spinulosa MAT*. Our starting point is the *MAT1-1-1*/*MAT1-2-3* fusion adjacent to *MAT1-2* in *T*. *reesei* strain QM6a (Fig 1). This strain is capable of mating with *H*. *jercorina MAT1-1* field isolate CBS999.97 [26]. We speculate that the fused ORF resulted from a misalignment of parental *MAT* chromosomes (*i*.*e*., between regions at the 3’ end of *MAT1-2-3* and within *MAT1-1-1*) during meiosis (S11A Fig). Indeed, a putative recombination site can be identified for this event (S11B Fig).

To achieve the *C*. *spinulosa* Cs23 *MAT1-1*;*MAT1-2* structure, we need to postulate that at least three different crossover events occurred in crosses between the putative ancestors of *C*. *spinulosa* (S12 Fig). In the first (S12A Fig), an unequal crossover would occur as described above, resulting in a *MAT1-2* progeny carrying a fused *MAT1-1-1*/*MAT1-2-3* gene (S12A Fig, asterisk) and a *MAT1-1-1* progeny, as described in S11A Fig for *Trichoderma*. In this cross, we assume that DR_1_ is included in the partial *MAT1-1-1* sequence. In the second crossover (S12B Fig), we postulate a similar event on the other side of DR_1_, resulting in a *MAT1-1* progeny (S12B Fig, asterisk). The third crossover would be between progeny of the first and second events (S12A and S12B Fig, asterisks) and would yield a progeny carrying the *MAT1-1*;*MAT1-2* structure present in *C*. *spinulosa* Cs23 (S12C Fig). Considering the high frequency of recombination (in the form of gene conversion) within the unusually large *MAT* locus (>100 kb) in the human pathogenic fungus *Cryptococcus neoformans* [38,39], this inference warrants further investigation.

### Conclusions

Our data clearly support a mechanism in which a DR-mediated looping out of *MAT1-2-1* occurs during mitotic growth (i.e., premeiotic) resulting in a strain with two different types of nuclei (genotypes *MAT1-1*;*MAT1-2* and *MAT1-1*) within a common cytoplasm in self-fertile *C*. *spinulosa*. Subsequently, in sexual reproduction, recognition and karyogamy occur between these two types of nuclei, and meiotic progeny are produced that segregate 1:1 for parental nucleus type [11]. Those with *MAT1-1*;*MAT1-2* are self-fertile and large, while those with *MAT1-1* only are self-sterile and smaller. We argue, therefore, that *C*. *spinulosa* is not primarily homothallic as are true homothallic species (e.g., *F*. *graminearum*), but employs a heterothallic mating strategy at the level of the nucleus (not cell), as previously suggested [19]. The *MAT1-2* deletion and imbalance of nuclear types are key to unidirectional mating-mode alteration. This hypothesis can also be applied to explain unidirectional mating-type alteration in *C*. *fimbriata* and *S*. *trifoliorum* [20,21]. Our study is the first to confirm, by *MAT* manipulation, that a chromosomal looping out-based mechanism underpins irreversible unidirectional mating-type alteration in filamentous fungi. Additionally, our results expand the repertoire of molecular mechanisms underlying homothallism and evolution of *MAT* loci in fungi.

## Methods

### Fungal strains, culture conditions, and staining of nuclei

*C*. *spinulosa* strains Cs23 and Cs27, which are self-fertile and self-sterile respectively, were kindly provided by Dr. John R. S. Fincham (S1 Fig) in 1997 and were used as the WT strains in this study. Transgenic strains derived from Cs23 and Cs27 mentioned in this study are listed in S1 Table. The WT and transgenic strains were maintained on potato dextrose agar (PDA; Difco Laboratories, Detroit, MI, USA) and stored in 20% glycerol at –70°C. Sexual development was induced on cornmeal agar (CMA, Difco) medium, as described previously [40]. Because of mitotic instability of Cs27-derived transformants carrying *MAT1-2-1* at the *MAT1-1* locus, ability to self was assayed with mycelia taken directly from transformation plates. For genomic DNA extraction, each strain was grown in 50 mL PD broth at 25°C for 72 h on a rotary shaker (150 rpm).The self-fertile *F*. *graminearum* WT strain Z3643 and its self-sterile transgenic *MAT1-1-1*-deletion strain [25], were maintained on PDA. Sexual development was induced on carrot agar, as previously described [25,41]. Fungal nuclei were stained with 4′,6-diamidino-2-phenylindole (DAPI) (Invitrogen, Carlsbad, CA) and examined as previously described [42].

### Nucleic acid manipulations, primers, and qPCR

To isolate genomic DNA, fungal strains grown in PD broth medium for 4 days at 25°C were harvested and lyophilized, as described previously [43]. DNA gel blots were hybridized with biotinylated DNA probes prepared using the BioPrime DNA labeling system (Invitrogen, Carlsbad, CA, USA) and developed using the BrightStar BioDetect Kit (Ambion, Austin, TX, USA). Other general procedures for nucleic acid manipulation were performed as described previously [44]. Total RNA was extracted using the Easy-Spin Total RNA Extraction Kit (iNtRON Biotechnology, Seongnam, Korea) and first-strand cDNA was synthesized from total RNA using ReverTra Ace qPCR RT Master mix (Toyobo, Osaka, Japan). All PCR primers (S2 Table) used in this study were synthesized by the Bioneer Corporation (Chungwon, Korea), diluted to 100 μM in sterilized water, and stored at −20°C. Quantitative real-time PCR (qPCR) was performed with SYBR Green Super Mix (Bio-Rad, Hercules, CA, USA) using first-strand cDNA synthesized from total RNA or genomic DNA from strains grown on PDA for 5 days. Amplification efficiencies of all genes were determined as described previously [45]. Gene expression was measured in three biological replicates from each time point. Statistical analysis was performed by ANOVA by Duncan's multiple range test. The *C*. *spinulosa EF1A* gene was used as endogenous control for data normalization [45].

### Construction of transforming DNA or plasmids

The DNA construct for deletion of DR_1_ from strain Cs23 was created using the double-joint (DJ) PCR procedure, as described previously [46]. To delete the DR_1_ region, the 5′- and 3′-flanking regions of the DR_1_ sequence (Fig 3) were amplified using the primer pairs CoHo5F/CoHo5RT and CoHo3FT/CoHo3R, respectively, and these were fused to a hygromycin B resistance gene cassette (*hygB*) amplified from pBCATPH [47] using primers hygB-For and hygB-Rev. The resulting PCR products were used as template for the final PCR to generate the gene deletion, using the primers CoHo5N/pUH-BC/H3 and CoHo3N/pUH-H2. Similarly, the DNA constructs for the generation of the transgenic strain TC27G-1 (S1 Table) were created as described above. Two DNA regions at the 3’ end of *MAT1-1-3* corresponding to nucleotide positions 11,702 in the Cs27 *MAT* and 15,291 in the Cs23 *MAT* locus, respectively, which start at the 3’ end of *MAT1-1-3*, were amplified by primer sets CoHoG5F/CoHoG5RT and CoHoG3FT/CoHoG3R, respectively, and fused to the geneticin resistance gene (*gen*) cassette using the primers CoHoG5N/GenForN and CoHoG3N/GenRenN.

For insertion of *MAT1-2* into Cs27, two different plasmid DNAs (pMAT2 and pM2M1, Fig 5), both carrying *MAT1-2-1*, were constructed. For pMAT2, the 5.3 kb-region carrying the entire *MAT1-2-1* ORF and its 5’ flank (1.0 kb) was amplified from Cs23 and put into the pGEMT vector, followed by *hygB* insertion at a *Sal*I site in pGEMT, as described in pM2M1. The 8.4-kb DNA region carrying *MAT1-2*-*1* and all three *MAT1-1* genes, which was amplified from Cs23, was inserted into the pGEMT vector (Promega), followed by the insertion of the *hygB* cassette at a *Sal*I site in pGEMT, resulting in pM2M1. For heterologous expression of Cs23 *MAT1-1-1* in a *MAT1-1-1-*deletion strain of *F*. *graminearum*, the entire ORF of each *MAT1-1-1* version amplified from Cs23 [*MAT1-1-1*L and *MAT1-1-1* with primer pairs Cs27forE/Cs23revE and Cs27forE/Cs27revE2 (S2 Table), respectively] was fused to DNA (~ 1 kb) both 5' and 3' of the *F*. *graminearum MAT1-1-1* (annotated as FGSG_08892.3 in the *F*. *graminearum* genome database) as described above [46]. The 5′- and 3′-flanking regions of the *F*. *graminearum MAT1-1-1* sequence were amplified using the primer pairs FgM1-1-1F5/FgM1-1-1Rt5Cs27 and Fgmat1-1-1Rt5Cs23 or FgM1-1-1Ft3Cs27 /FgM1-1-1R3Cs27 (S2 Table), respectively, and these were fused to either Cs23 *MAT1-1-1*L or *MAT1-1-1*, followed by the final PCR amplification using nested primers FgM1-1-1FNCs27 and FgM1-1-1RNCs27 (S2 Table).

### Sequencing of *MAT* loci

Nucleotide sequences of Cs23 and Cs27 *MAT* loci (Fig 1) were obtained using conventional PCR amplification followed by a combination of TAIL-PCR and inverse PCR amplification as chromosome walking strategies (S2 Fig). First, a 270-bp fragment of the High Mobility Group (HMG) box of *MAT1-2-1* was amplified from genomic DNA of Cs23 using degenerate HMG primers (NcHMG1 and NcHMG2 [48], designated P1 and P2 in S2 Table, S2 Fig), after which TAIL-PCR [49] was performed to obtain sequence beyond the HMG box using combinations of arbitrary and specific primers, such as P4 [49]/P3 for the 3’ flank and P5 [49]/P6 for the 5’ flank. To recover the rest of the *MAT* region, we employed an inverse PCR strategy, as described previously [31]. Genomic DNA from Cs23 was digested with *Bam*HI, *Sph*I, *Cla*I, *Nhe*I, and *Sac*II, self-ligated, and used as a template with appropriate primer pairs as shown in S2 Fig. Subsequently, sequencing was extended using primers corresponding to previously determined sequences. Sequence assembly (21.9 kb) revealed that the *C*. *spinulosa* Cs23 *MAT* chromosome included *MAT1-2-1*, three *MAT1-1* genes and additional ORFs (S2 Fig, Fig 1). Similarly, a total of 18.3 kb of the *MAT* chromosomal region was recovered from Cs27 using the inverse PCR strategy and conventional PCR with the primers derived from the Cs23 *MAT* region, as shown in S2 Fig. Nucleotide sequences were assembled (Cs23, Cs27: GenBank accessions # KY624604 and # KY624603) and analyzed using the DNASTAR software package (DNAStar Inc., Madison, WI, USA). BLAST [50] searches were performed against the NCBI/GenBank databases.

### Fungal transformation

The *C*. *spinulosa* Cs23, Cs27, and T10 strains were transformed using a polyethylene glycol (PEG)-mediated transformation procedure newly developed in this study. For preparation of young mycelia for protoplasting, approximately 50 agar blocks (3 × 3 mm) from a 7-day old PDA culture of a strain were inoculated into 100 ml PD broth and incubated for 24 h on a shaker (200 rpm). Mycelia, harvested by centrifugation, were re-suspended in 100 ml fresh PD broth in a 500 ml flask and incubated for an additional 24 h under the same conditions. Note that *C*. *spinulosa* was unable to produce asexual spores (conidia) under all growth conditions examined. Young mycelia were recovered by centrifugation and suspended in 25 ml osmoticum (0.7 M KCl) containing 800 mg of lysing enzyme from *Trichoderma harzianum* and 8 mg of cellulase from *Trichoderma* sp. (Sigma). The suspension was incubated for 3 h at 30°C on a shaker (50 rpm). Protoplasts, which were collected by filtering through three layers of cheesecloth followed by centrifugation, were suspended in 10 ml STC (1.2 M Sorbitol, 10 mM Tris-HCl pH 8, and 50 mM CaCl_2_). All other transformation steps using protoplasts were performed as described previously [43]. Two genes conferring resistance to hygromycin B (*hygB*) or geneticin (*gen*) were used as selectable markers for fungal transformation in this study.

To determine if Cs23 *MAT1-1-1* L is functional, we inserted it and *MAT1-1-1*, separately, into a *F*. *graminearum* Δ*MAT1-1-1* strain [25]. PCR constructs were directly added to protoplasts of the *F*. *graminearum* Δ*MAT1-1-1* strain along with the plasmid DNA pSSK660 carrying a geneticin resistance gene (*gen*) [51] (S8A Fig). As a positive control, the *F*. *graminearum MAT1-1-1* region (carrying the entire *MAT1-1-1* along with its 5' and 3' flanking regions) was added using the same strategy.

## Supporting information

S1 Table *C*. *spinulosa* strains used in this study. Strain name, a brief description, and *MAT* genotype and phenotype are described.
(DOCX)

S2 Table Primers used in this study. Primer names, sequence, position in overall sequence and references are listed.
(DOCX)

S3 Table Comparison of features associated with *MAT1-2-1* elimination. *C*. *spinulosa*, *S*. *trifoliorum*, and *C*. *fimbriata MAT* structural features are compared.
(DOCX)

S1 Fig 1997 note from the late John Fincham to corresponding author BGT. The note traces the origin of the Cs23 and Cs27 strains and mentions a 1952 paper [11] describing mating phenomena in this fungus. JF subsequently sent the strains to BGT.
(TIF)

S2 Fig PCR strategies for amplifying the *MAT* regions and beyond from *C*. *spinulosa* Cs23 and Cs27 strains. All primers indicated are listed in S2 Table. See Fig 1 legend for gene explanations.
(TIF)

S3 Fig Cartoon of looping out of the MAT1-2-1 region. Numbers refer to positions in the 21.9 kb of Cs23 sequence generated (S2 Fig). Note that the 3’ end of the *MAT1-1-1*L fragment (green box), is lost in the recombination event.
(PPTX)

S4 Fig Colony growth of transgenic strains derived from Cs27 after several growth cycles. Control Cs23 and Cs27 in the first rows were grown on PDA without hygromycin B. Typical candidates generated by integration of *MAT1-2-1* (see Fig 5), were grown on PDA or PDA amended with hygromycin B. **(A).** Shows a T27M1M2a-type transformant after three cycles. **(B).** Shows a T27M1M2b-type transformant after three cycles. Note in both cases, ability to grow on hygromycin medium is lost by cycle 3.
(TIF)

S5 Fig *MAT* structures and DNA gel blots of DNA of progeny from the T10 by Cs27 outcrosses. **(A).** *MAT* structure in T10, Cs23 and Cs27-type strains. **(B).** Gel blots of *Sac*I-digested genomic DNAs from strains of all categories represented in **(A)**. Hybridization with *MAT1-1-1*. **(C and D).** As in **(B)**, but hybridized with DR and *MAT1-2-1* probes, respectively. From left, Cs23, Cs27, T10, TCs27Gen1, Cs23-type progeny (12, 26, 28, 30, and 60), T10-type progeny (48 and 102), and Cs27-type progeny (10, 25, and 70). All strains are listed in S1 Table; progeny are prefixed with “P”. Sizes (in kb) are indicated to the left of the gel.
(TIF)

S6 Fig Nucleotide sequences and positions of primers used for qPCR experiments to distinguish the *MAT1-1-1*L fragment from the intact copy of *MAT1-1-1*. PCR primer sequences are shown in S2 Table, results in S7 Fig. Primer pair qCs27M1-1-1R/qCs23M1-1-1F, is specific to *MAT1-1-1*L, while primer pair qCs27M1-1-1R/qCs27M1-1-1F amplifies *MAT1-1-1* from strains carrying either *MAT1-1-1* or *MAT1-1-1*L.
(TIF)

S7 Fig *MAT1-1-1*L is expressed. **(A).** Relative expression levels of individual *MAT* transcripts determined by qPCR on total RNA (A). Amplification level of *MAT1-2-1* in Cs23 was used as a reference (set to 1). The same letter above bars indicates no significant difference. Primer pair qCs27M1-1-1R/qCs23M1-1-1F, specific to *MAT1-1-1*L, amplified the predicted 138 bp fragment from both Cs23 and T10, while the primer pair qCs27M1-1-1R/ qCs27M1-1-1F, amplified the predicted 106 bp fragment from both Cs23 and Cs27. Note that the 106 bp fragment was not amplified from RNA of strain T10 because one of two repeats had been deleted. See S6 Fig for primer positions. Other primer pairs were as described in Fig 7. **(B).** Relative amounts of individual *MAT* genes determined by qPCR on genomic DNA.Amplification level of *MAT1-2-1* in Cs23 was set to 1 as reference. See **(A)** for primer details.
(TIF)

S8 Fig Heterologous expression of *MAT1-1-1*L in a *F*. *graminearum* strain lacking *MAT1-1-1* (FgΔ*MAT1-1-1*). **(A).** Scheme for the insertion of *MAT1-1-1*L or *MAT1-1-1* from Cs23 (designated Cs*MAT1-1-1*) into the FgΔ*MAT1-1-1* strain by co-transformation with pSSK660 carrying the geneticin resistance gene (*gen*). The transgenic FgΔ*MAT1-1-1* strains carrying either Cs*MAT1-1-1* or Cs*MAT1-1-1*L at an ectopic position are designated as FgΔ*MAT1-1-1*::Cs*MAT1-1-1* and FgΔ*MAT1-1-1*::Cs*MAT1-1-1*L, respectively. Drug resistance phenotypes are in parentheses. **(B).** Perithecium formation.: *F*. *graminearum* self-fertile WT Z3643 strain, FgΔ*MAT1-1-1*: a *MAT1-1-1-*deletion strain of Z3643, FgΔ*MAT1-1-1*::Fg*MAT1-1-1*: a control add-back strain of FgΔ*MAT1-1* carrying an intact copy of Fg*MAT1-1-1* at an ectopic position, FgΔ*MAT1-1-1*::Cs*MAT1-1-1*-16: a FgΔ*MAT1-1-1*::Cs*MAT1-1-1* strain, and FgΔ*MAT1-1-1*::Cs*MAT1-1-1*L-35: a FgΔ*MAT1-1-1*::Cs*MAT1-1-1*L strain. Scale-bar = 500 μm. **(C).** Average diameter of perithecia formed on carrot agar cultures. Perithecial sizes from two independent transformants carrying *C*. *spinulosa MAT1-1-1* (6, 13) or *MAT1-1-1*L (35, 29) are shown. The same letters on each bar represent no significant difference.
(TIF)

S9 Fig **DAPI staining of nuclei in ascospores from a self of strain Cs23 (A-B), and an outcross between strains T10 and Cs27 (C-D).** DIC images corresponding to DAPI fluorescence images are shown in B and D, respectively. Large and small ascospores within an ascus are designated with L and S, respectively. Asci containing different numbers of nuclei (ranging from those in the diploid zygote to those in complete tetrads with 16 nuclei) are indicated by arrows. Scale bars = 50μm.
(TIF)

S10 Fig **High magnification of DAPI stained nuclei in ascospores from a self of Cs23 (A and C).** Corresponding DIC images are shown in B and D, respectively. Large and small ascospores are designated with L and S, respectively. Several ascospores each containing 2–3 nuclei are indicated by arrows. Nuclei in small ascospores were difficult to stain in mature spores (C, D). Scale bars = 20 μm.
(TIF)

S11 Fig Proposed distribution of nuclei in *C*. *spinulosa* strains described in this study. Self-fertile Cs23 and Cs23-type progeny carry two different versions of *MAT* (*MAT1-1* only and *MAT1-1*;*MAT1-2*) in a common cytoplasm. The latter are in the minority. Self-sterile strains (Cs27, T10, and T10-type progeny) carry nuclei containing a single version of *MAT* but architecture can vary. Self-sterile T27M12a-E5 carries both *MAT1-1* and *MAT1-2*, but likely not closely linked to each other.
(TIF)

S12 Fig Possible genetic mechanism for the origin of *MAT1-2* in the *Trichoderma reesei* QM6a strain. **(A).** A model for the evolution of the fused *MAT1-1-1*:*MAT1-2-3* gene. We propose that a recombination event occurred *via* misalignment of paired *MAT* chromosomes in *T*. *reesei* ancestors [represented by *T*. *virens* Gv29-8 (a *MAT1-2* parent) and *H*. *jecorina* (a *MAT1-1* parent), respectively] resulting in the fused gene in extant strain QM6a. **(B)**. A possible crossover point for the model shown in **(A)** in the actual nucleotide sequences of *T*. *virens* Gv29-8 and *H*. *jecorina* CBS 999.97 strains. Nucleotide sequences conserved between strains are indicated by the same colors.
(TIF)

S13 Fig Possible scenario for the evolutionary history of *MAT* locus organization in *C*. *spinulosa*. To achieve the *MAT1-1*;*MAT1-2* structure present in *C*. *spinulosa* Cs23, we assume at least three different unequal crossing over events occurred in putative heterothallic ancestors (as in S12 Fig). In the first cross **(A)**, a crossover would occur in a manner similar to that described in S12 Fig, resulting in a *MAT1-2* progeny (asterisk) carrying a fused gene consisting of a 3’ portion of *MAT1-1-1* and *MAT1-2-3*. In this case, a possible crossover point would be to the right of the DR (red bar) in the *MAT1-1* parent chromosome, leaving the DR_1_ associated with the *MAT1-1-1* fragment in the fused protein. In the second case **(B)**, a similar event might occur, but with a possible crossover site to the left of the DR in the *MAT1-1* parent chromosome. This would result in a *MAT1-1* progeny (asterisk) carrying a truncated *MAT1-1-1* gene that includes the DR sequence at its 3’ end. The third crossover **(C)** would occur between the *MAT1-2* and *MAT1-1* progeny generated from the **(A)** and **(B)**. If the crossover site were between a region 3’ of *MAT1-2-1* 3’ and a region 3’ of *MAT1-1-1*, this would yield a progeny carrying *MAT* organization of *C*. *spinulosa* Cs23 (Figs 1 and 2). Refer to Fig 1 for gene organization on the *MAT* chromosome.
(TIF)
