# Supplementary material for: Self-fertility in Chromocrea spinulosa is a consequence of direct repeat-mediated loss of MAT1-2, subsequent imbalance of nuclei differing in mating type, and recognition between unlike nuclei in a common cytoplasm
Source: PLoS Genet. 2017 Sep 11;13(9):e1006981. doi: 10.1371/journal.pgen.1006981 (PMC5608430; doi:10.1371/journal.pgen.1006981)
Supplement: S1 Table — Strain name, a brief description, and MAT genotype and phenotype are described. (DOCX) [file pgen.1006981.s001.docx]

S1 Table. *C. spinulosa* strains used in this study

| **Strain** | **Brief description** | ***MAT* genotype & mating phenotype** |
| --- | --- | --- |
| C23 | *C. spinulosa* wild type | *MAT1-1*;*MAT1-2*^a^ & *MAT1-1*; Self-fertile |
| Cs27 | *C. spinulosa* wild type | *MAT1-1*; Self-sterile |
| T10 | DR_1_ deletion strain in Cs23-1 | *MAT1-1*;*MAT1-2*; Self-sterile; hygB^R^ |
| P11, P17, P48, P102 | T10-type progeny from the outcross between T10 and Cs27 | *MAT1-1*;*MAT1-2*^a^; Self-sterile; hygB^R^ |
| P12, P26, P28, P30, P60 | Cs23-type progeny from the outcross between T10 and Cs27 | *MAT1-1*;*MAT1-2*^a^; Self-fertile; hygB^R^ |
| P3, P10, P25, P70, | Cs27-type progeny from the outcross between T10 and Cs27 | *MAT1-1*; Self-sterile |
| TC27G-1 | Cs27 transformant carrying *gen* in *MAT1-1-3* 3’ flank | *MAT1-1*; Self-sterile; gen^R^ |
| T27M12a-H1 | Cs27 transformant carrying *MAT1-2-1* near the resident *MAT1-1* locus | *MAT1-2* & *MAT1-1*^b^; Self-sterile; hygB^R^ |
| T27M12b-H3 | Cs27 transformant carrying *MAT1-2-1* near the resident *MAT1-1* locus | *MAT1-2* & *MAT1-1*^b^; Self-sterile; hygB^R^ |
| T27M12a-E5 | Cs27 transformant carrying *MAT1-2-1* at an ectopic position | *MAT1-2* & *MAT1-1*^b^; Self-sterile; hygB^R^ |
| T27M12a-E25^c^ | Cs27 transformant carrying *MAT1-2-1* at an ectopic position | *MAT1-2* & *MAT1-1*^b^; Self-sterile; hygB^R^ |
| FgΔ*MAT1-1-1*:: Cs*MAT1-1-1*-16 | a *F. graminearum* Δ*MAT1-1-1* strain carrying *MAT1-1-1* from Cs23 | *MAT1-2* & *MAT1-1*; Self-sterile^d^; hygB^R^;gen^R^ |
| FgΔ*MAT1-1-1*:: Cs*MAT1-1-1*L-35 | a *F. graminearum* Δ*MAT1-1-1* strain carrying *MAT1-1-1*L from Cs23 | *MAT1-2* & *MAT1-1;* Self-sterile^d^; hygB^R^;gen^R^ |
| ^a^ closely linked *MAT1-2* and *MAT1-1* loci carrying *MAT1-2-1, MAT1-1-1, MAT1-1-2,* and *MAT1-1-3* (Figs. 1, 2).  ^b^see Fig. 5.  ^c^ produced perithecia without asci/ascospores  ^d^capable of promoting perithecia formation | | |
