# Supplementary material for: Self-fertility in Chromocrea spinulosa is a consequence of direct repeat-mediated loss of MAT1-2, subsequent imbalance of nuclei differing in mating type, and recognition between unlike nuclei in a common cytoplasm
Source: PLoS Genet. 2017 Sep 11;13(9):e1006981. doi: 10.1371/journal.pgen.1006981 (PMC5608430; doi:10.1371/journal.pgen.1006981)
Supplement: S2 Table — Primer names, sequence, position in overall sequence and references are listed. (DOCX) [file pgen.1006981.s002.docx]

| **S2 Table. Primers used in this study** | | |  |  |
| --- | --- | --- | --- | --- |
| **Name** | **Sequence (5' to 3')** | **Length (bp)** | **Positions** | **References** |
| P1 | CCYCGYCCYCCYAAYGCNTAYAT | 23 | 9,549-9,571 | [46] |
| P2 | CGNGGRTTRTARCGRTARTNRGG | 23 | 9,818-9,796 | [46] |
| P3 | CTGGGACGAGCCTGGAATCAAGAATC | 26 | 9,703-9,728 | this study |
| P4 | NGTCGASWGANAWGAA | 16 |  | [47] |
| P5 | WGTGNAGWANCANAGA | 16 |  | [47] |
| P6 | GGCAGCTTTGACCATATTGTGACGA | 25 | 9,611-9,587 | this study |
| P7 | CGTACTGAAAAGGATCTGCTCTCGGAAAT | 29 | 10,678-10,650 | this study |
| P8 | TGGGACATGAAACGTTGAGAAAATAGGAGA | 29 | 10,810-10,839 | this study |
| P9 | TTCAACAAACATCACGGCAAAGGTA | 24 | 6,135-6,159 | this study |
| P10 | TCTAAGCCTGGCCCACGGATTGATTCTGA | 29 | 9,943-9,915 | this study |
| P11 | CAGAAGTCGAATGTTGGTATGCGTGATGA | 29 | 12,682-12,710 | this study |
| P12 | CTGCATGCCCAAGGTCCACA | 20 | 6,205-6,186 | this study |
| P13 | ACTCCCGGTCTCTTGGTGGTTAG | 23 | 15,563-15,585 | this study |
| CP1 | TGTCGTGATGGTAATGGAGAAAAC | 24 | 9,363-9,386 | this study |
| CP2 | GATTGATTCTGAGCTCCGTTTTG | 23 | 9,926-9,904 | this study |
| CP3 | GAAAATTGCGTCCCAGGTTGTCA | 23 | 10,103-10,125 | this study |
| CP4 | CGATGAACGCCAGTAGCAATAGC | 23 | 10,602-10,580 | this study |
| CP5 | TGTGGGTCTGGATAAATAAACTGC | 24 | 10,359-10,382 | this study |
| CP6 | AAGTCTCCCTCTCCTATTTTCTCAACG | 27 | 10,848-10,822 | this study |
| CoHo5F | GTCGACGCATCGTATCGGGACAC | 24 |  | this study |
| CoHo5RT | CAGGTACACTTGTTTAGAGTGTTTGGCCATCAGGAGAATGAAG | 43 |  | this study |
| CoHo3FT | TCAATATCATCTTCTGTCGGACTTGCTTCGATTCAGTTGCTAT | 43 |  | this study |
| CoHo3R | ATTTATTTCTCCAGTCGTTCCCCATCCTCA | 30 |  | this study |
| CoHo5N | TTTACTAACATTGCGTCAGCCTGGAAC | 27 |  | this study |
| CoHo3N | TCGATCTTTCACTTCAACTTCACTACTGGA | 30 |  | this study |
| CoHo-G5F | TCTCTTCTCTTCATCGTTCATCACAGT | 27 |  | this study |
| CoHo-G5Rt | CAGGTACACTTGTTTAGACCAGTGGCCGACGCTTCCTACAG | 41 |  | this study |
| CoHo-G3Ft | TCAATATCATCTTCTGTCGCTGGTCCTGCACACGTGAAACAT | 42 |  | this study |
| CoHo-G3R | TGAATACATGCTGGGACACGAA | 22 |  | this study |
| CoHo-GFN | CTGGAAACAAAAGCTCATTAGAACC | 25 |  | this study |
| CoHo-GRN | CTACCCCGGAAAGAGAACCAACGACA | 26 |  | this study |
| qCs27M1-1-1F | AAGCGACACATTTGATCTTGACA | 23 |  | this study |
| qCs27M1-1-1R | GTCTACAATTACGCATCCACCAC | 23 |  | this study |
| qCs23M1-1-1F | CGTAAAAGGGTAAATGCAGTATAT | 24 |  | this study |
| qCsmat1-1for7 | GCTGGGATAGAATTGGAGGGAGTC | 24 |  | this study |
| qCsmat1-1rev7 | CCGTACTGAAAAGGATCTGCTCTC | 24 |  | this study |
| qCsmat1-2for6 | AAATTGGTCCTTGCTCTCCTCTGG | 24 |  | this study |
| qCsmat1-2rev6 | TCTGCGACGTTATTGACTGCTGTG | 24 |  | this study |
| qCsmat1-3for4 | AGGATCCACCGTGAATACTT | 20 |  | this study |
| qCsmat1-3rev4 | GCGAAAAACTGGCGATACAA | 20 |  | this study |
| qCsmat2for4 | GCACCGCGTTTCTTTATTG | 19 |  | this study |
| qCsmat2rev4 | CATTAGGAGGGCGTGGGATTTTG | 23 |  | this study |
| FgM1-1-1F5 | GTGTGGGGCAGGGCGGAGTG | 20 |  | this study |
| FgM1-1-1Rt5cs27 | GTTCTACTACACTTTCTAAGTGATGAACTATTTGCACATGTCGGGC | 46 |  | this study |
| FgM1-1-1Ft3cs27 | TCCGCTCTCGACGTCATAGTTAAATTGCAGAGGTGTGTAAGG | 42 |  | this study |
| FgM1-1-1R3cs27 | TGGATTGGGGGCTATACTGACGAG | 24 |  | this study |
| FgM1-1-1FNcs27 | TCAGGTCAAAACAATCCTCACTATG | 25 |  | this study |
| FgM1-1-1RNcs27 | CATCTTTTTCGGTGGCTTGGGCTATT | 26 |  | this study |
| Fgmat1-1-1Rt5cs23 | CTGGTAAAAACTTTTTGAGTGATGAACTATTTGCACATGTCGGGC | 45 |  | this study |
| Cs27revE2 | TTAGAAAGTGTAGTAGAACTATTC | 24 |  | this study |
| Cs27forE | ATGACGTCGAGAGCGGAATTGG | 22 |  | this study |
| Cs23revE | TCAAAAAGTTTTTACCAGTTGGTCT | 25 |  | this study |
| Gen forN | TGCTGCTTGGACAAATGAACG | 21 |  | this study |
| Gen revN | CCGCTTGGGTGGAGAGGCTATT | 22 |  | this study |
| hygB-For | CTCTAAACAAGTGTACCTGTGC | 22 |  | this study |
| hygB-Rev | CGACAGAAGATGATATTGAAGG | 22 |  | this study |
| pUH-BC/H3 | GCTGCTCCATACAAGCCAACC | 21 |  | this study |
| pUH-BC/H2 | CGTTATGTTTATCCTGCACTTTGC | 24 |  | this study |

Y = C or T; R = A or G; S = C or G; W = A or T; N = A, G, C or T.
