# Supplementary material for: Self-fertility in Chromocrea spinulosa is a consequence of direct repeat-mediated loss of MAT1-2, subsequent imbalance of nuclei differing in mating type, and recognition between unlike nuclei in a common cytoplasm
Source: PLoS Genet. 2017 Sep 11;13(9):e1006981. doi: 10.1371/journal.pgen.1006981 (PMC5608430; doi:10.1371/journal.pgen.1006981)
Supplement: S3 Table — C. spinulosa, S. trifoliorum, and C. fimbriata MAT structural features are compared. (DOCX) [file pgen.1006981.s003.docx]

| **S3 Table.** **Comparison of features associated with *MAT1-2-1* elimination** | | | |
| --- | --- | --- | --- |
| **Feature** | **Species** | | |
|  | *C. spinulosa* | *S. trifoliorum* | *C. fimbriata* |
| Repeat size | 115 | 146 | 260 |
| Large *MAT1-1-1* | 1140 | 677 | NA |
| Small *MAT1-1-1* | 229 | 601 | NA |
| Large *MAT1-1-1* expressed? | yes | yes | NA |
| *MAT1-2* deletion  bp eliminated | 3,349 | 2966 | 3581 |
| Recombination event | pre-meiotic | meiosis | pre-meiotic |
| # ascospores | 16 | 8 | 8 |
| Alpha box | intact | intact | intact |
| Karyogamy | between a *MAT1-1*;  *MAT1-2* and *MAT1-1* nucleus | not mentioned | implied between a *MAT1-1*;*MAT1-2* and *MAT1-1* nucleus |
| Self-sterile:self-fertile  segregation ratio | 1:1 | 1:1 | 1:1 to 9:1 |
| Reference | this work | Xu et al., 2016 | Wilken et al., 2014 |

NA, not applicable; ND, not known
