# Supplementary figures and images for: Self-fertility in Chromocrea spinulosa is a consequence of direct repeat-mediated loss of MAT1-2, subsequent imbalance of nuclei differing in mating type, and recognition between unlike nuclei in a common cytoplasm
Source: PLoS Genet. 2017 Sep 11;13(9):e1006981. doi: 10.1371/journal.pgen.1006981 (PMC5608430; doi:10.1371/journal.pgen.1006981)

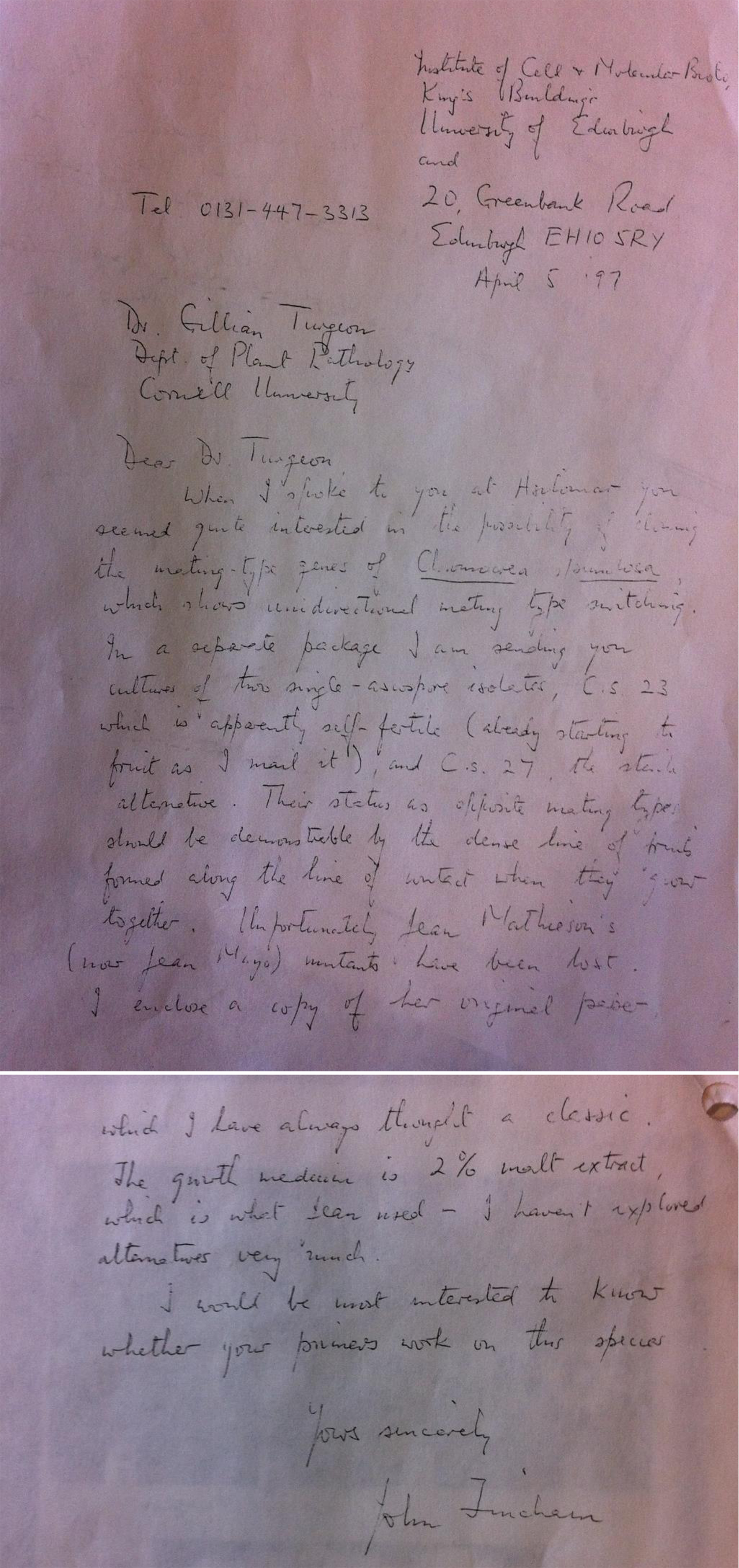

Supplement: S1 Fig — The note traces the origin of the Cs23 and Cs27 strains and mentions a 1952 paper [11] describing mating phenomena in this fungus. JF subsequently sent the strains to BGT. (TIF) [file pgen.1006981.s004.tif]

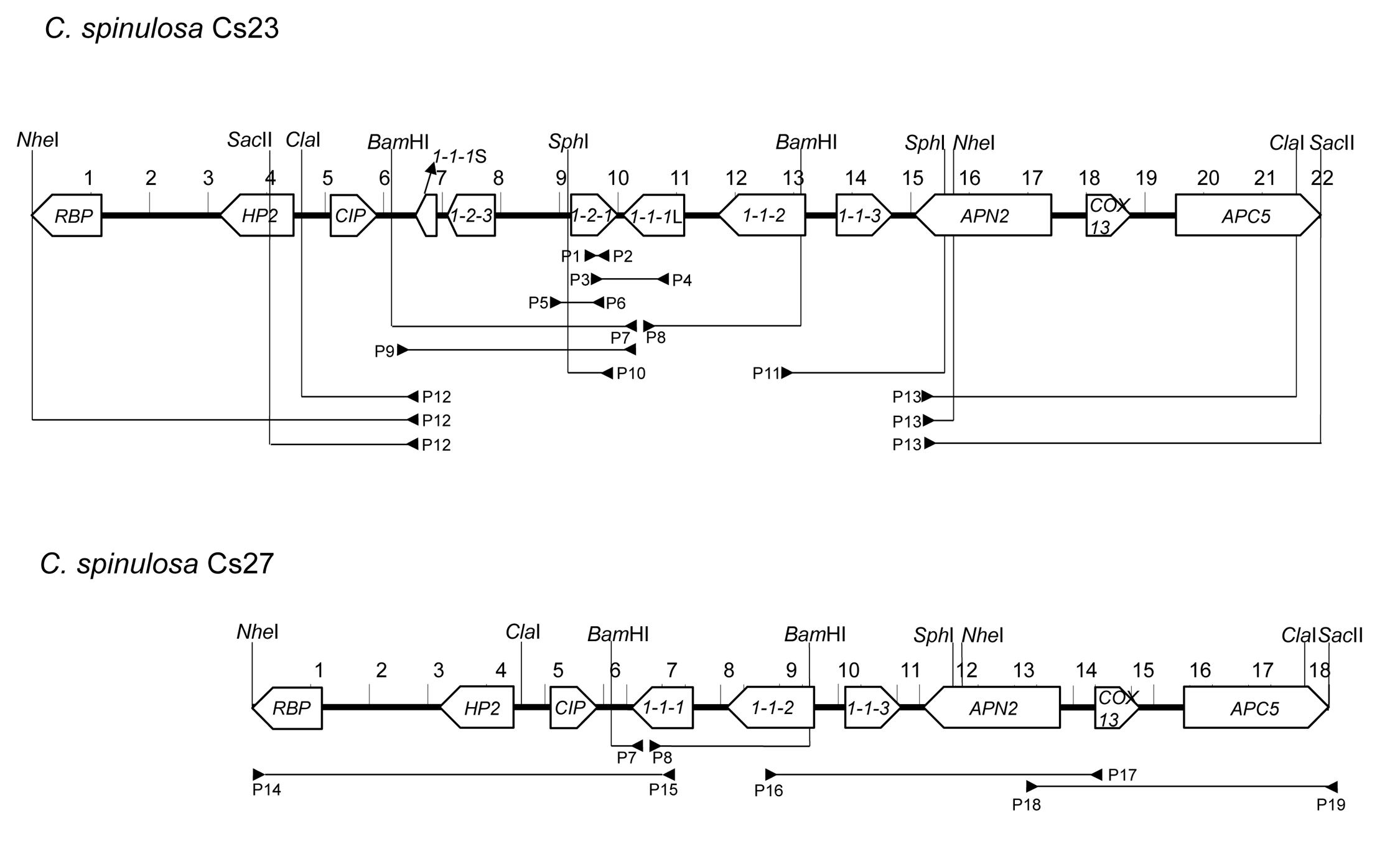

Supplement: S2 Fig — All primers indicated are listed in S2 Table. See Fig 1 legend for gene explanations. (TIF) [file pgen.1006981.s005.tif]

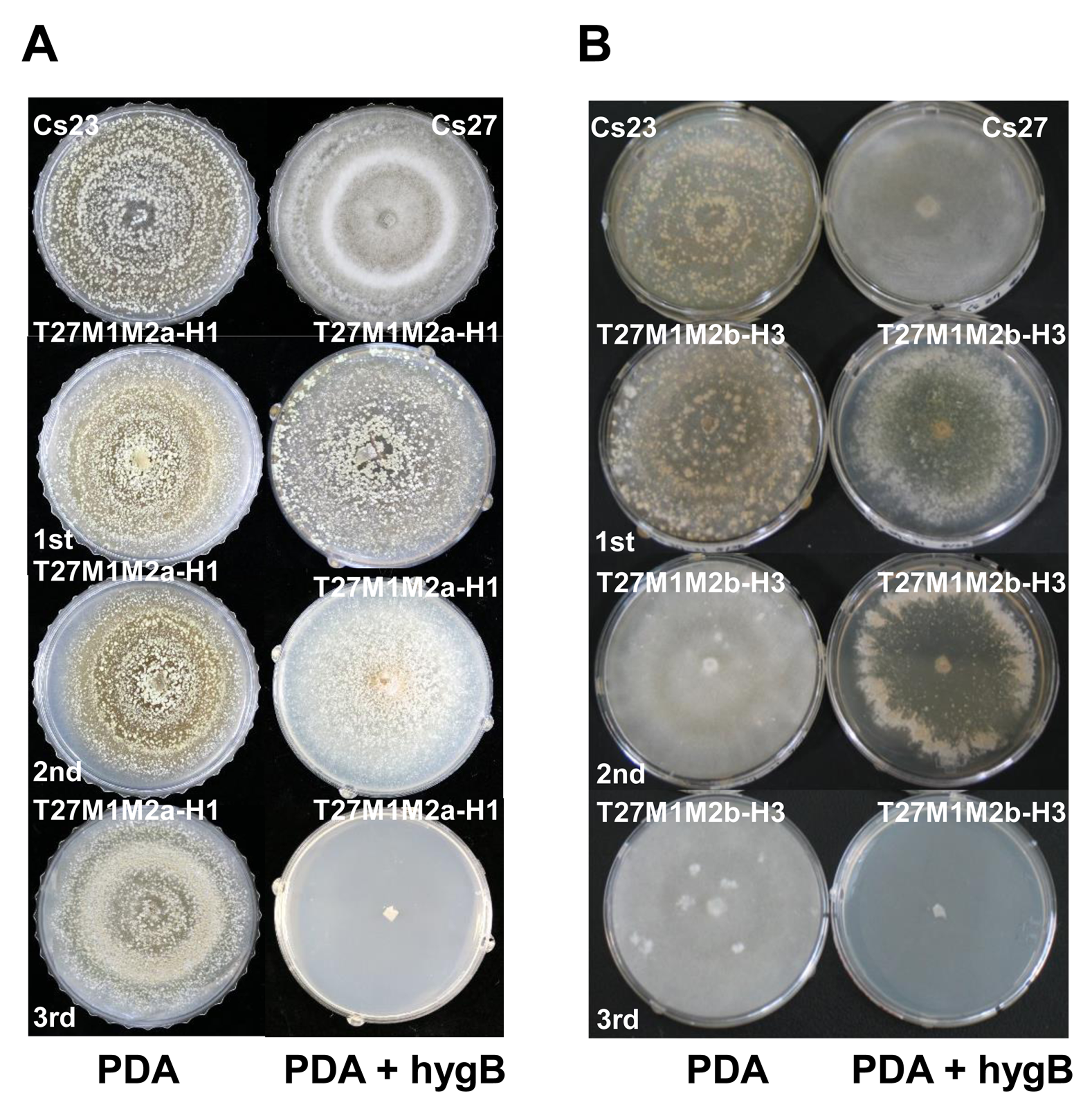

Supplement: S4 Fig — Control Cs23 and Cs27 in the first rows were grown on PDA without hygromycin B. Typical candidates generated by integration of MAT1-2-1 (see Fig 5), were grown on PDA or PDA amended with hygromycin B. (A). Shows a T27M1M2a-type transformant after three cycles. (B). Shows a T27M1M2b-type transformant after three cycles. Note in both cases, ability to grow on hygromycin medium is lost by cycle 3. (TIF) [file pgen.1006981.s007.tif]

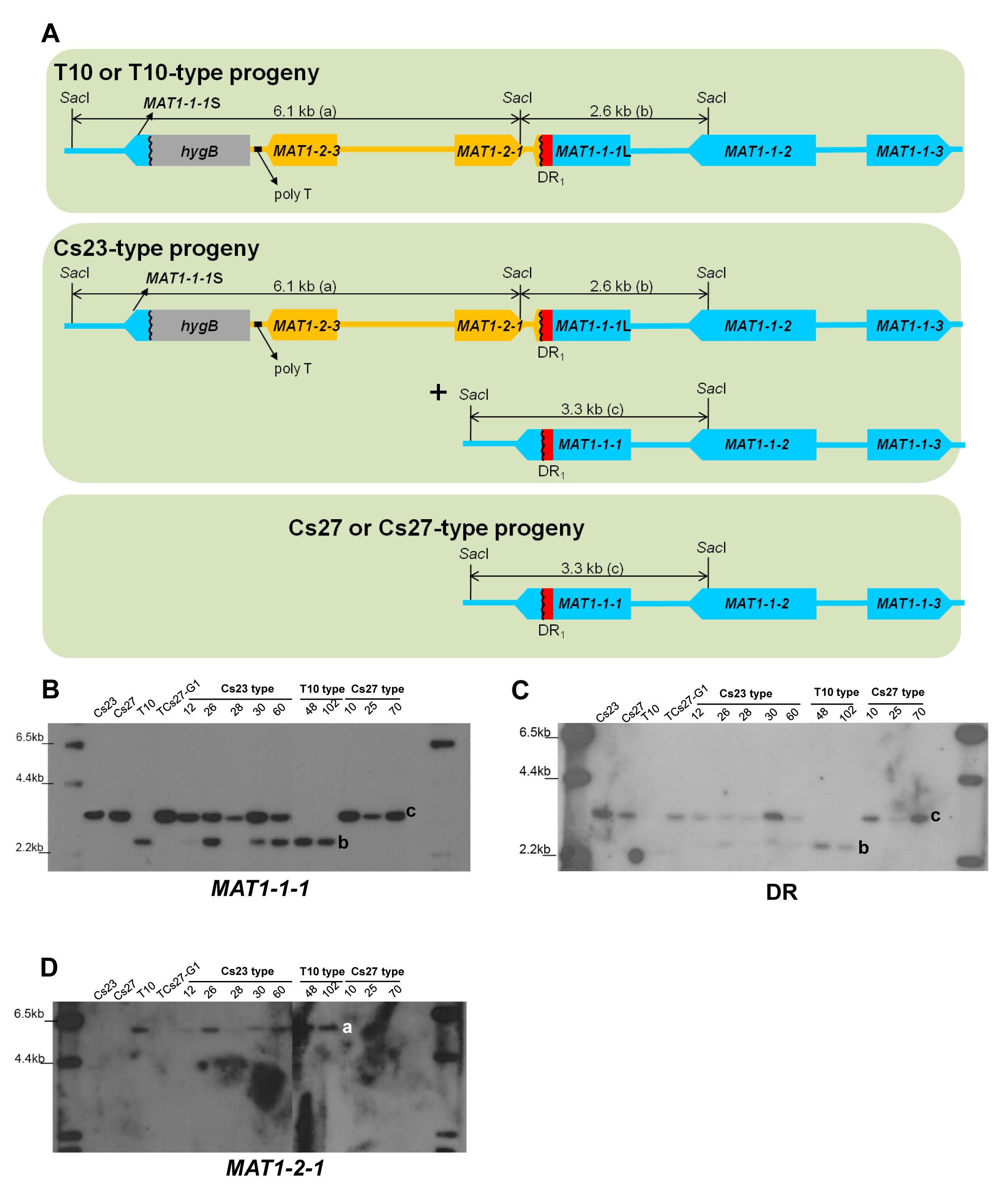

Supplement: S5 Fig — (A). MAT structure in T10, Cs23 and Cs27-type strains. (B). Gel blots of SacI-digested genomic DNAs from strains of all categories represented in (A). Hybridization with MAT1-1-1. (C and D). As in (B), but hybridized with DR and MAT1-2-1 probes, respectively. From left, Cs23, Cs27, T10, TCs27Gen1, Cs23-type progeny (12, 26, 28, 30, and 60), T10-type progeny (48 and 102), and Cs27-type progeny (10, 25, and 70). All strains are listed in S1 Table; progeny are prefixed with “P”. Sizes (in kb) are indicated to the left of the gel. (TIF) [file pgen.1006981.s008.tif]

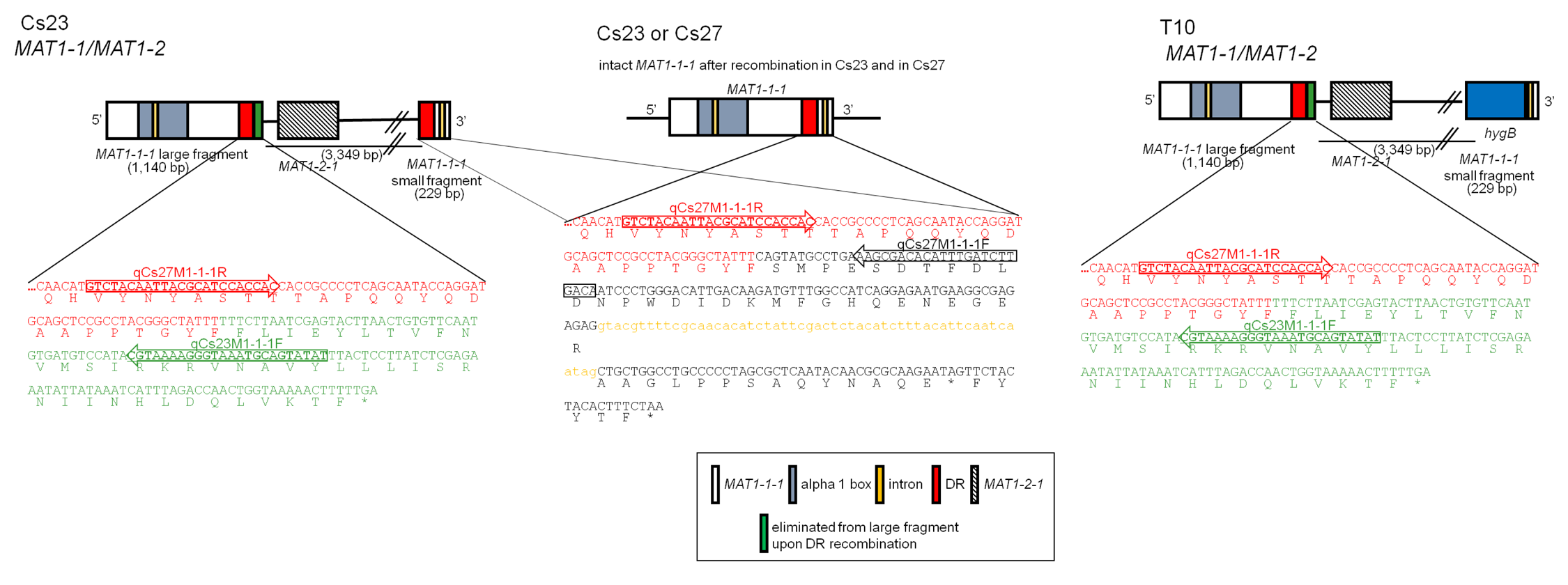

Supplement: S6 Fig — PCR primer sequences are shown in S2 Table, results in S7 Fig. Primer pair qCs27M1-1-1R/qCs23M1-1-1F, is specific to MAT1-1-1L, while primer pair qCs27M1-1-1R/qCs27M1-1-1F amplifies MAT1-1-1 from strains carrying either MAT1-1-1 or MAT1-1-1L. (TIF) [file pgen.1006981.s009.tif]

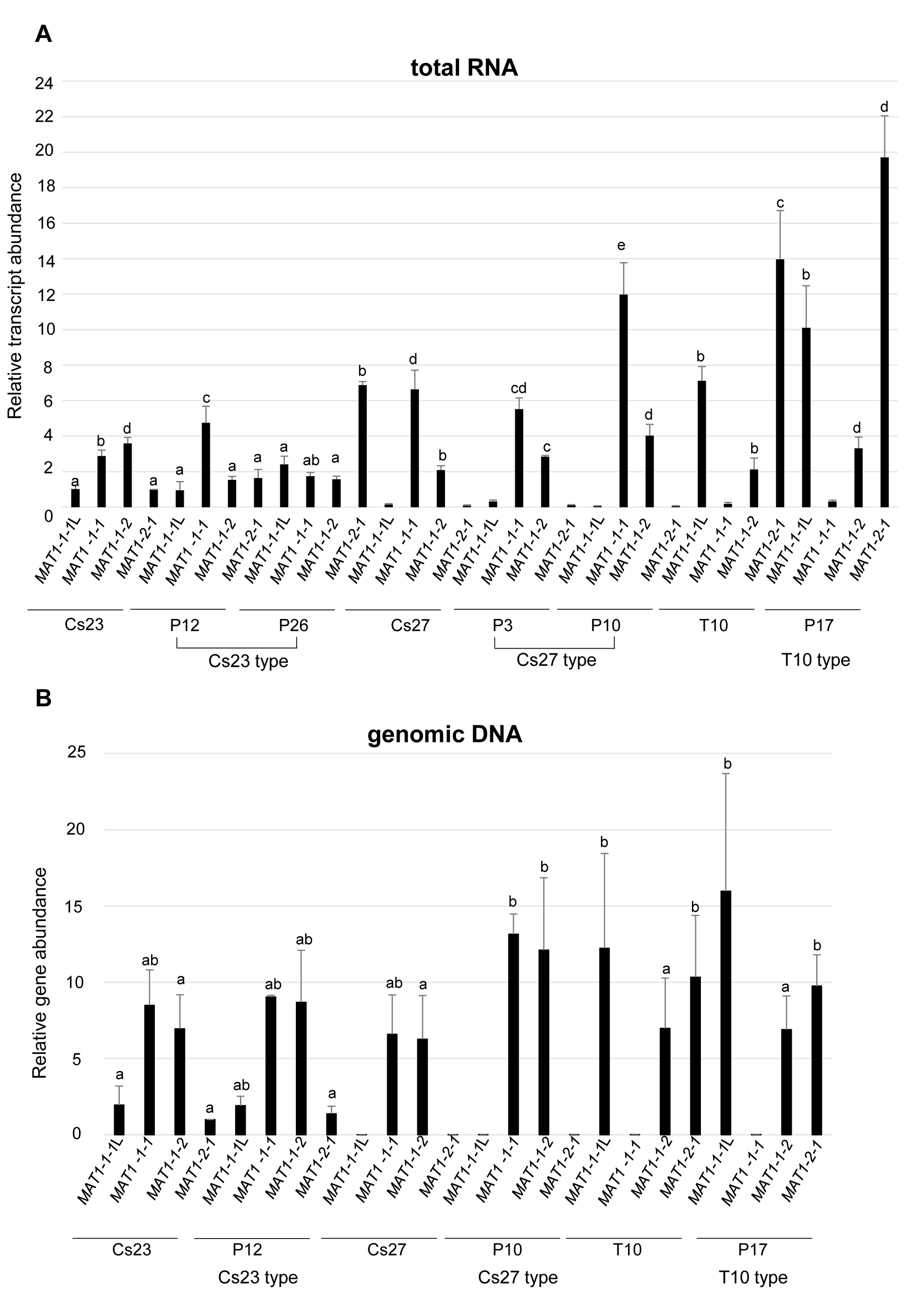

Supplement: S7 Fig — (A). Relative expression levels of individual MAT transcripts determined by qPCR on total RNA (A). Amplification level of MAT1-2-1 in Cs23 was used as a reference (set to 1). The same letter above bars indicates no significant difference. Primer pair qCs27M1-1-1R/qCs23M1-1-1F, specific to MAT1-1-1L, amplified the predicted 138 bp fragment from both Cs23 and T10, while the primer pair qCs27M1-1-1R/ qCs27M1-1-1F, amplified the predicted 106 bp fragment from both Cs23 and Cs27. Note that the 106 bp fragment was not amplified from RNA of strain T10 because one of two repeats had been deleted. See S6 Fig for primer positions. Other primer pairs were as described in Fig 7. (B). Relative amounts of individual MAT genes determined by qPCR on genomic DNA.Amplification level of MAT1-2-1 in Cs23 was set to 1 as reference. See (A) for primer details. (TIF) [file pgen.1006981.s010.tif]

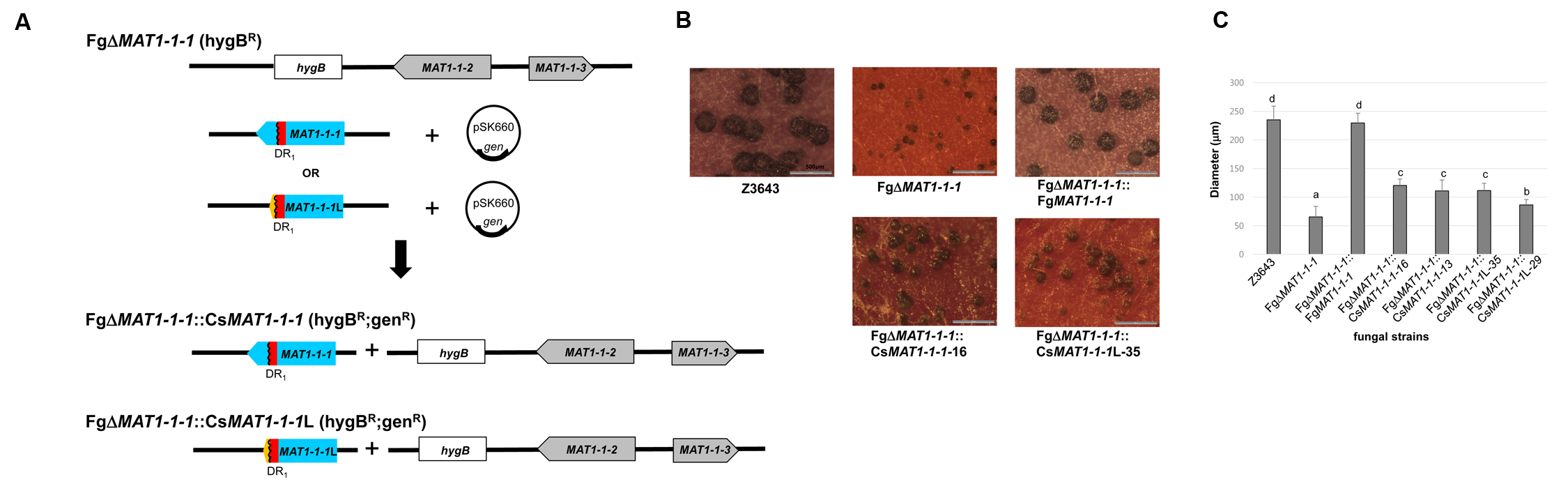

Supplement: S8 Fig — (A). Scheme for the insertion of MAT1-1-1L or MAT1-1-1 from Cs23 (designated CsMAT1-1-1) into the FgΔMAT1-1-1 strain by co-transformation with pSSK660 carrying the geneticin resistance gene (gen). The transgenic FgΔMAT1-1-1 strains carrying either CsMAT1-1-1 or CsMAT1-1-1L at an ectopic position are designated as FgΔMAT1-1-1::CsMAT1-1-1 and FgΔMAT1-1-1::CsMAT1-1-1L, respectively. Drug resistance phenotypes are in parentheses. (B). Perithecium formation.: F. graminearum self-fertile WT Z3643 strain, FgΔMAT1-1-1: a MAT1-1-1-deletion strain of Z3643, FgΔMAT1-1-1::FgMAT1-1-1: a control add-back strain of FgΔMAT1-1 carrying an intact copy of FgMAT1-1-1 at an ectopic position, FgΔMAT1-1-1::CsMAT1-1-1-16: a FgΔMAT1-1-1::CsMAT1-1-1 strain, and FgΔMAT1-1-1::CsMAT1-1-1L-35: a FgΔMAT1-1-1::CsMAT1-1-1L strain. Scale-bar = 500 μm. (C). Average diameter of perithecia formed on carrot agar cultures. Perithecial sizes from two independent transformants carrying C. spinulosa MAT1-1-1 (6, 13) or MAT1-1-1L (35, 29) are shown. The same letters on each bar represent no significant difference. (TIF) [file pgen.1006981.s011.tif]

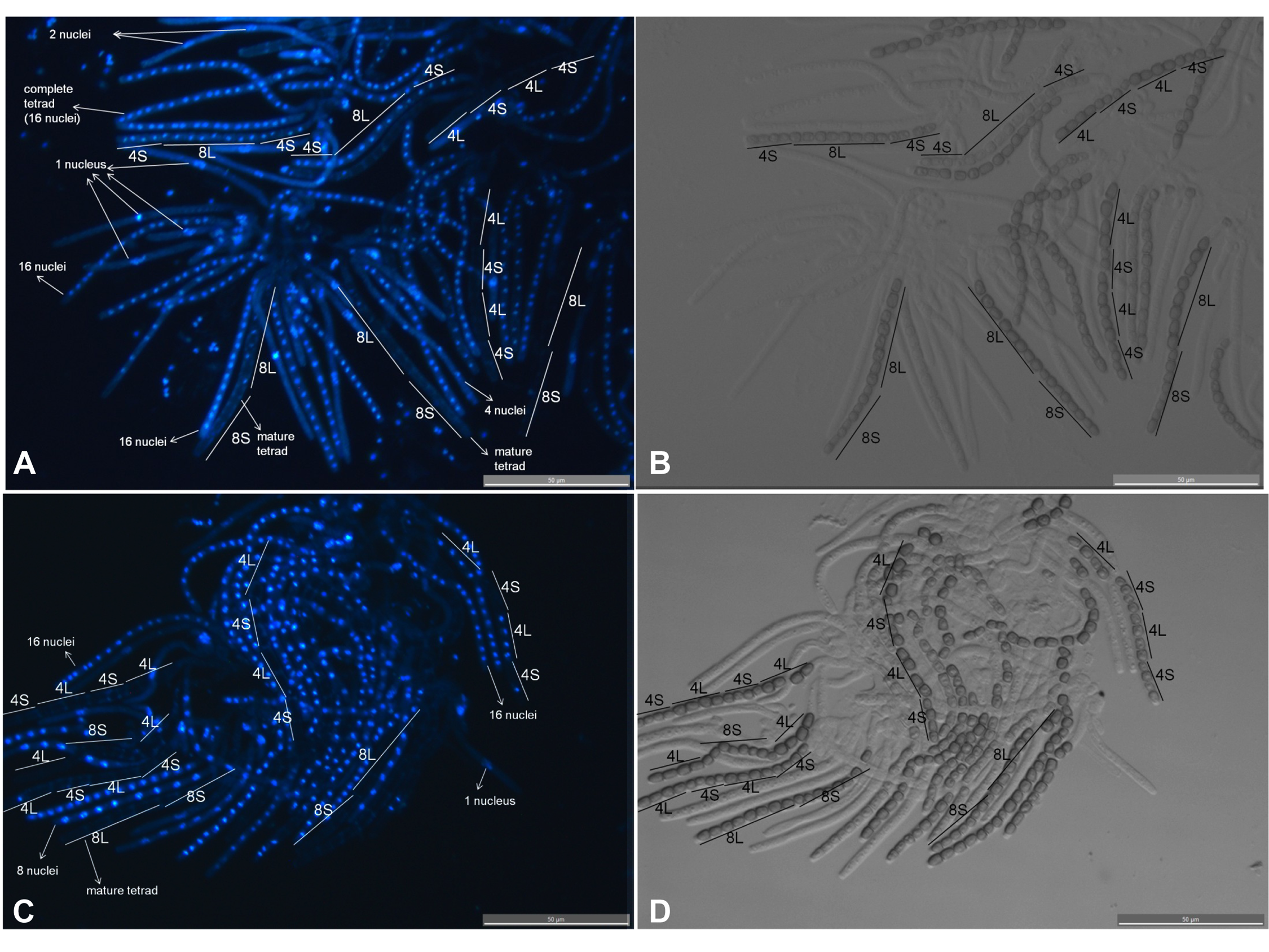

Supplement: S9 Fig — DAPI staining of nuclei in ascospores from a self of strain Cs23 (A-B), and an outcross between strains T10 and Cs27 (C-D). DIC images corresponding to DAPI fluorescence images are shown in B and D, respectively. Large and small ascospores within an ascus are designated with L and S, respectively. Asci containing different numbers of nuclei (ranging from those in the diploid zygote to those in complete tetrads with 16 nuclei) are indicated by arrows. Scale bars = 50μm. (TIF) [file pgen.1006981.s012.tif]

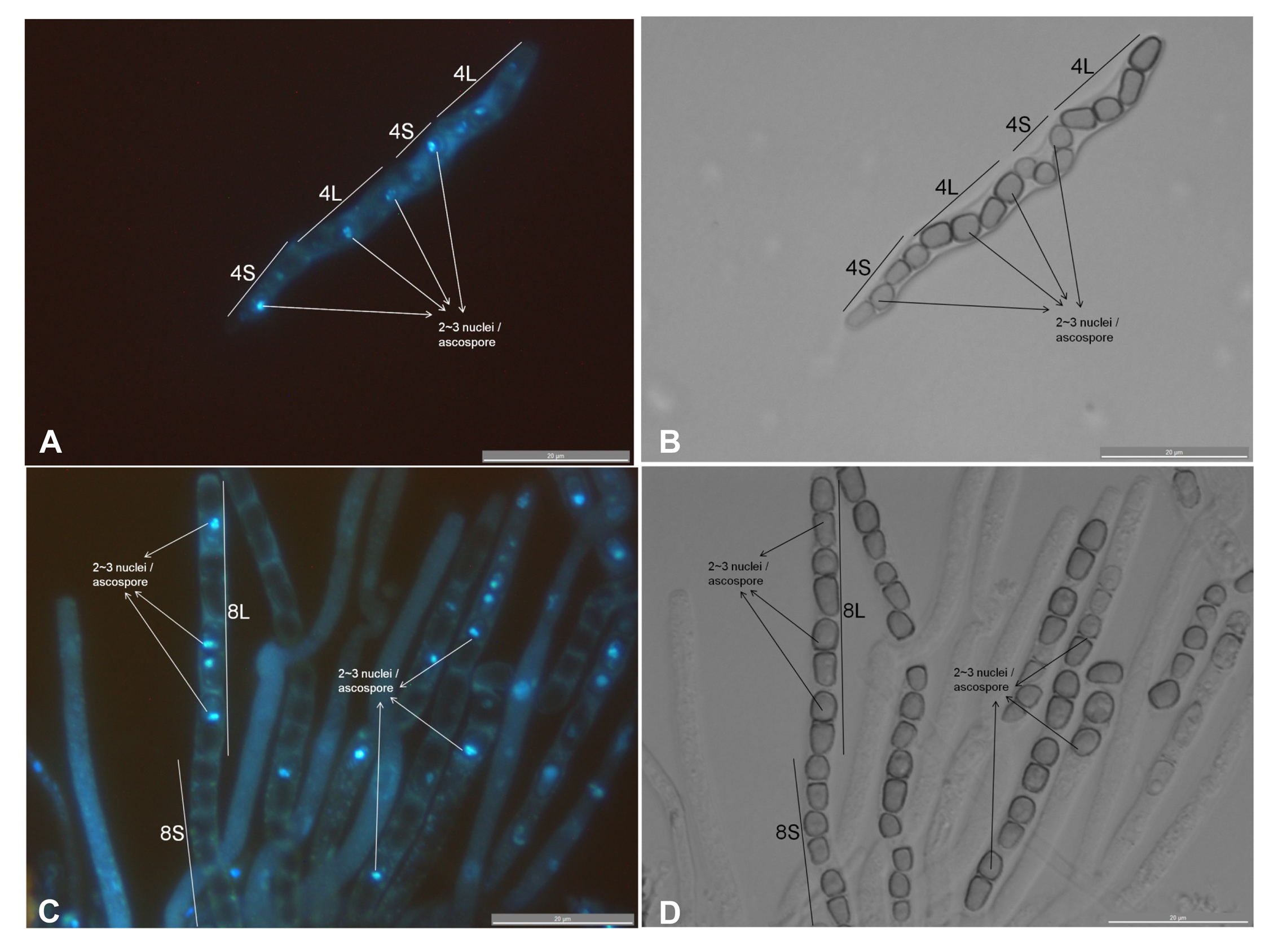

Supplement: S10 Fig — High magnification of DAPI stained nuclei in ascospores from a self of Cs23 (A and C). Corresponding DIC images are shown in B and D, respectively. Large and small ascospores are designated with L and S, respectively. Several ascospores each containing 2–3 nuclei are indicated by arrows. Nuclei in small ascospores were difficult to stain in mature spores (C, D). Scale bars = 20 μm. (TIF) [file pgen.1006981.s013.tif]

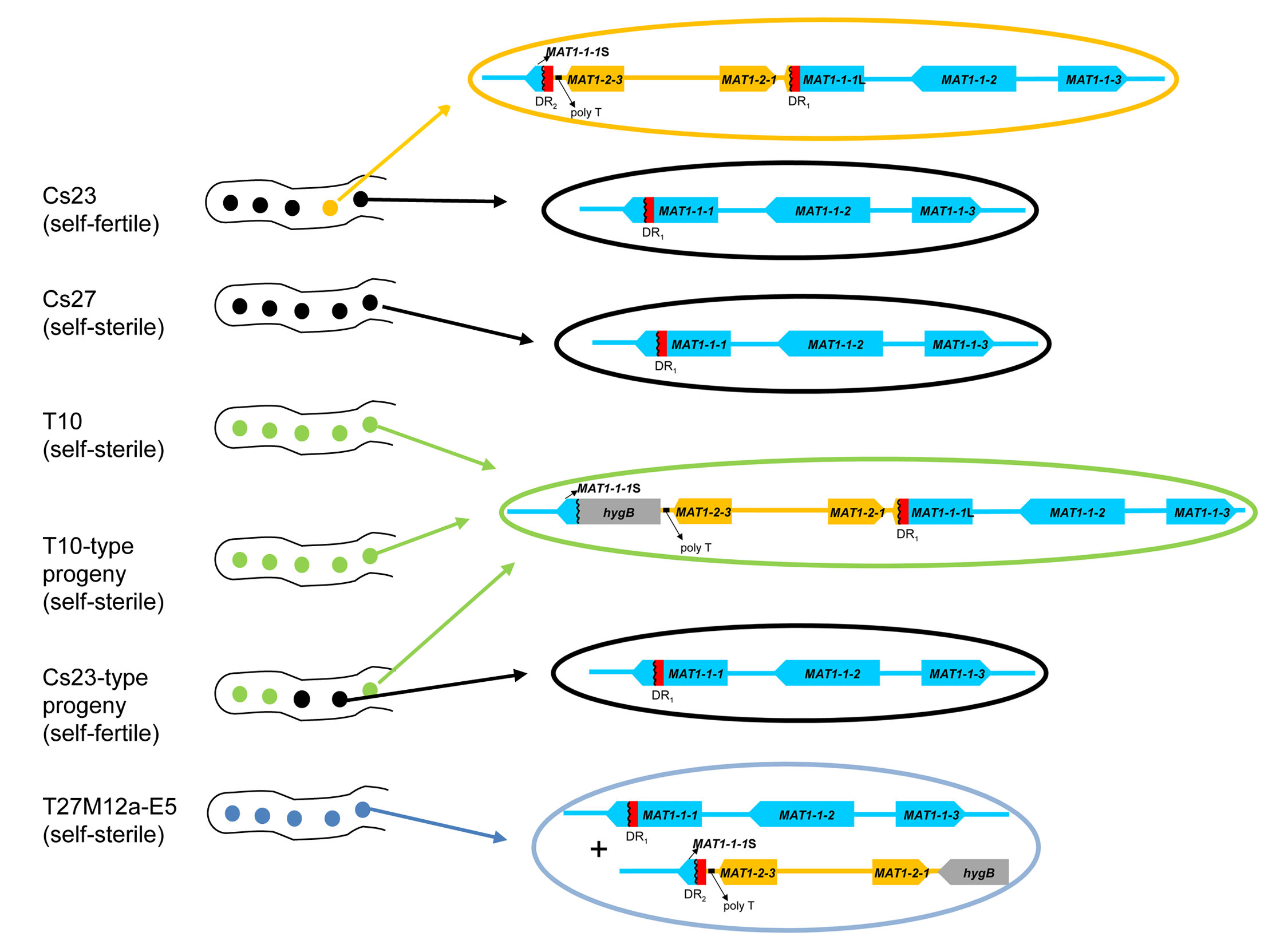

Supplement: S11 Fig — Self-fertile Cs23 and Cs23-type progeny carry two different versions of MAT (MAT1-1 only and MAT1-1;MAT1-2) in a common cytoplasm. The latter are in the minority. Self-sterile strains (Cs27, T10, and T10-type progeny) carry nuclei containing a single version of MAT but architecture can vary. Self-sterile T27M12a-E5 carries both MAT1-1 and MAT1-2, but likely not closely linked to each other. (TIF) [file pgen.1006981.s014.tif]

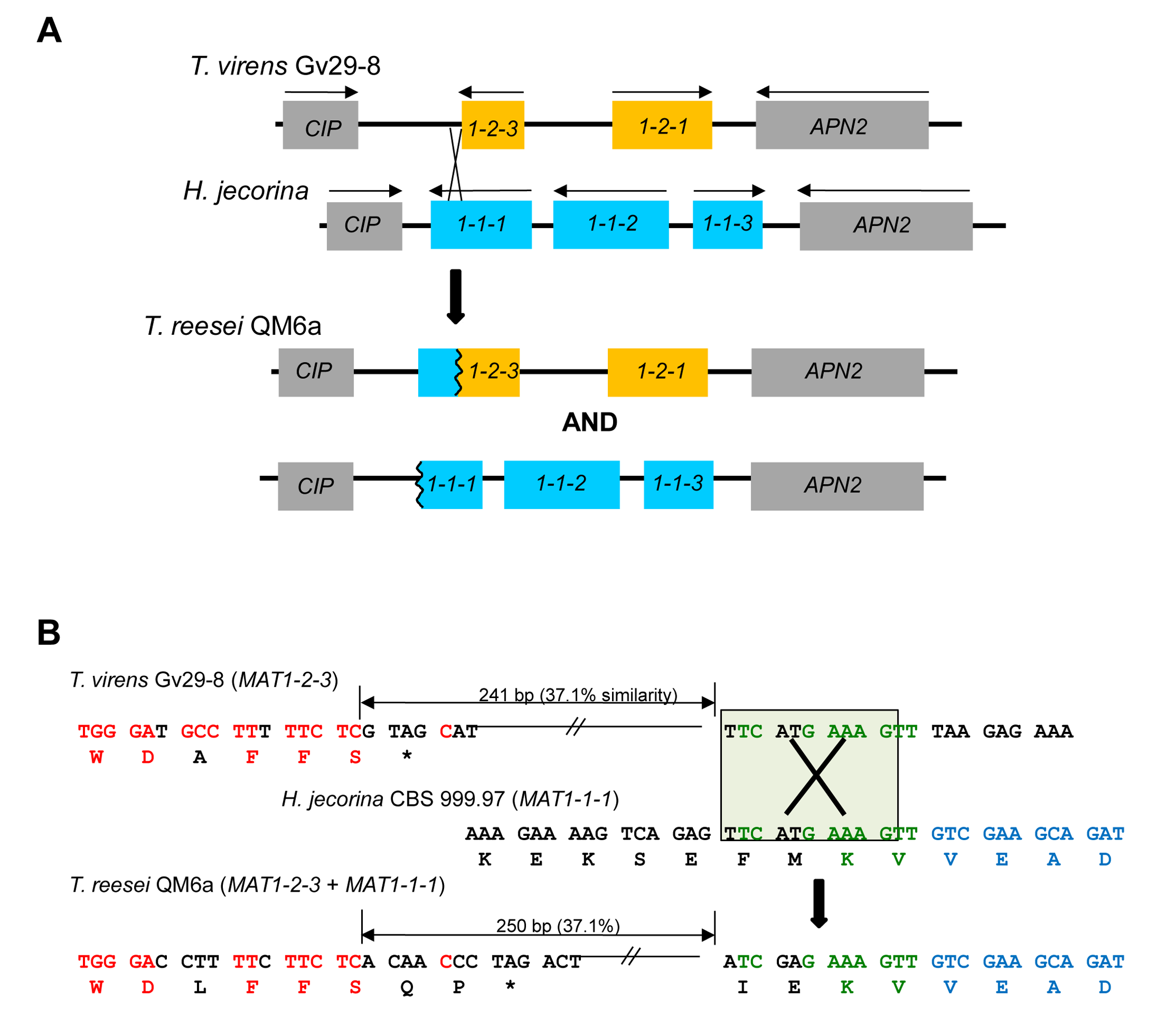

Supplement: S12 Fig — (A). A model for the evolution of the fused MAT1-1-1:MAT1-2-3 gene. We propose that a recombination event occurred via misalignment of paired MAT chromosomes in T. reesei ancestors [represented by T. virens Gv29-8 (a MAT1-2 parent) and H. jecorina (a MAT1-1 parent), respectively] resulting in the fused gene in extant strain QM6a. (B). A possible crossover point for the model shown in (A) in the actual nucleotide sequences of T. virens Gv29-8 and H. jecorina CBS 999.97 strains. Nucleotide sequences conserved between strains are indicated by the same colors. (TIF) [file pgen.1006981.s015.tif]

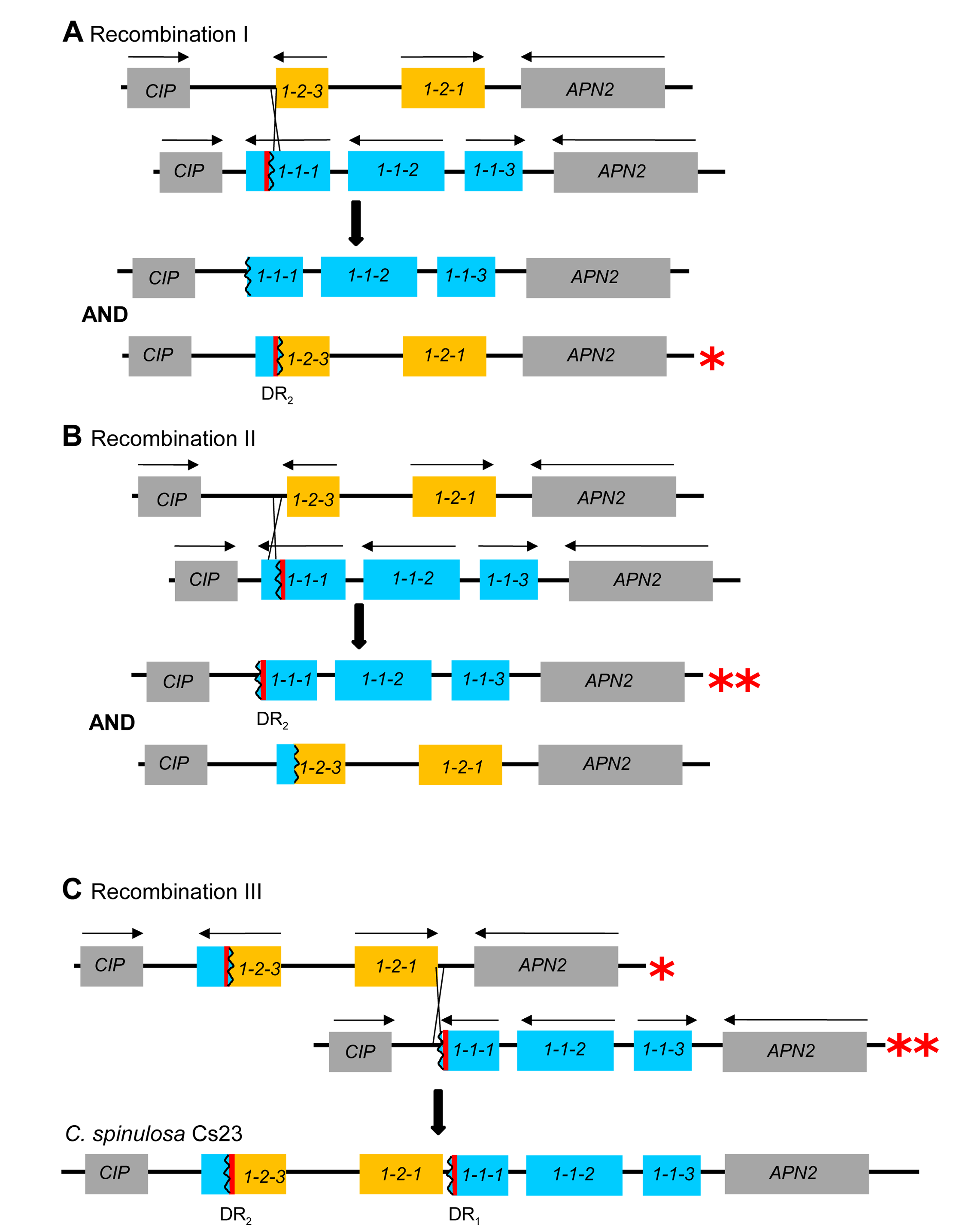

Supplement: S13 Fig — To achieve the MAT1-1;MAT1-2 structure present in C. spinulosa Cs23, we assume at least three different unequal crossing over events occurred in putative heterothallic ancestors (as in S12 Fig). In the first cross (A), a crossover would occur in a manner similar to that described in S12 Fig, resulting in a MAT1-2 progeny (asterisk) carrying a fused gene consisting of a 3’ portion of MAT1-1-1 and MAT1-2-3. In this case, a possible crossover point would be to the right of the DR (red bar) in the MAT1-1 parent chromosome, leaving the DR1 associated with the MAT1-1-1 fragment in the fused protein. In the second case (B), a similar event might occur, but with a possible crossover site to the left of the DR in the MAT1-1 parent chromosome. This would result in a MAT1-1 progeny (asterisk) carrying a truncated MAT1-1-1 gene that includes the DR sequence at its 3’ end. The third crossover (C) would occur between the MAT1-2 and MAT1-1 progeny generated from the (A) and (B). If the crossover site were between a region 3’ of MAT1-2-1 3’ and a region 3’ of MAT1-1-1, this would yield a progeny carrying MAT organization of C. spinulosa Cs23 (Figs 1 and 2). Refer to Fig 1 for gene organization on the MAT chromosome. (TIF) [file pgen.1006981.s016.tif]
