# Supplementary material for: Self-fertility in Chromocrea spinulosa is a consequence of direct repeat-mediated loss of MAT1-2, subsequent imbalance of nuclei differing in mating type, and recognition between unlike nuclei in a common cytoplasm
Source: PLoS Genet. 2017 Sep 11;13(9):e1006981. doi: 10.1371/journal.pgen.1006981 (PMC5608430; doi:10.1371/journal.pgen.1006981)
Supplement: S3 Fig — Numbers refer to positions in the 21.9 kb of Cs23 sequence generated (S2 Fig). Note that the 3’ end of the MAT1-1-1L fragment (green box), is lost in the recombination event. (PPTX) [file pgen.1006981.s006.pptx]

## Slide 1
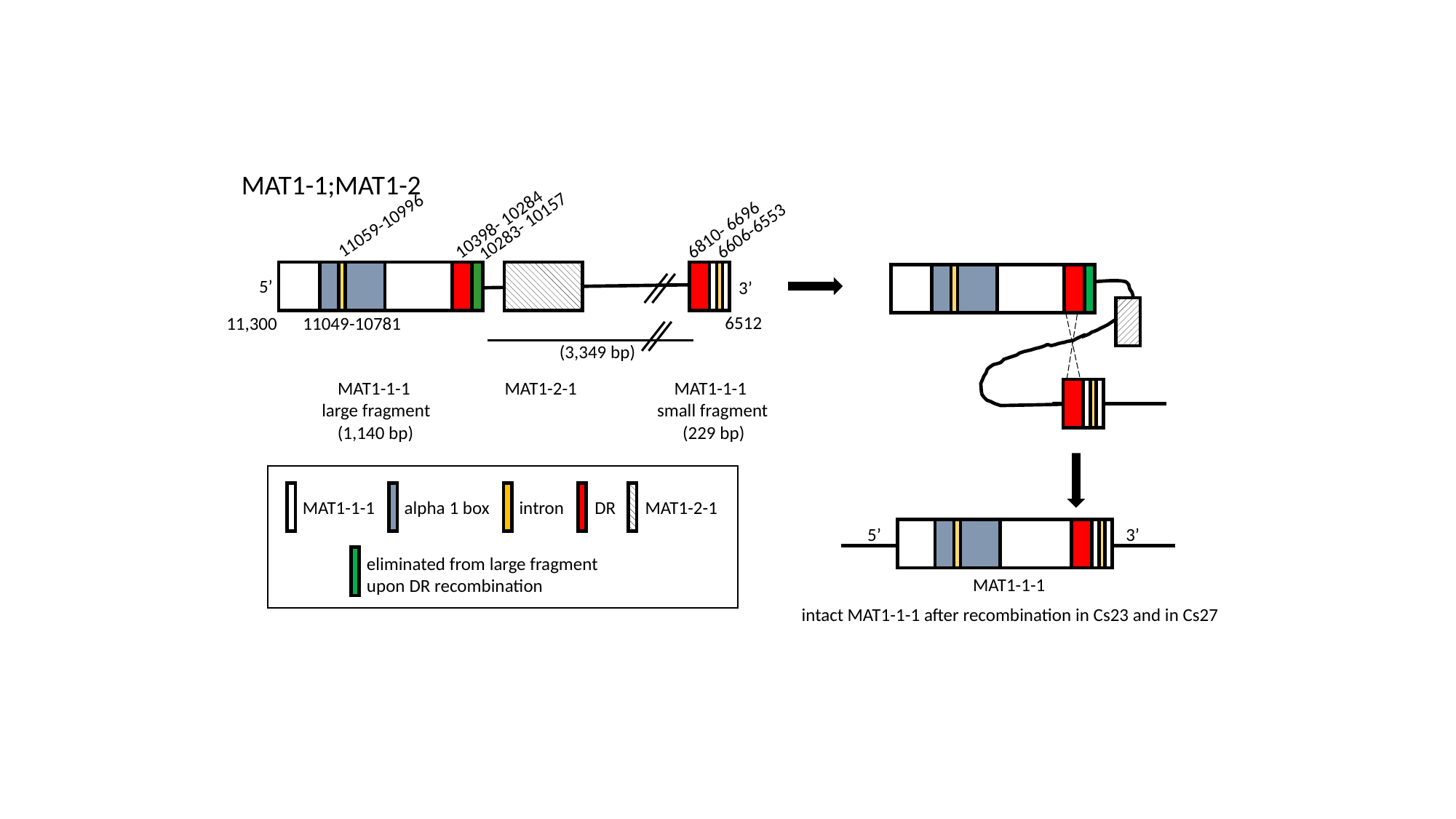

MAT1-1;MAT1-2
10398- 10284
11059-10996
6810- 6696
6606-6553
5’
3’
6512
11,300
11049-10781
(3,349 bp)
MAT1-1-1
large fragment
MAT1-2-1
MAT1-1-1
small fragment
(1,140 bp)
(229 bp)
MAT1-1-1
alpha 1 box
intron
DR
MAT1-2-1
5’
3’
eliminated from large fragment
upon DR recombination
MAT1-1-1
intact MAT1-1-1 after recombination in Cs23 and in Cs27
10283- 10157
